# Optineurin deficiency in mice contributes to impaired cytokine secretion and neutrophil recruitment in bacteria-driven colitis

**DOI:** 10.1242/dmm.020362

**Published:** 2015-08-01

**Authors:** Thean S. Chew, Nuala R. O'Shea, Gavin W. Sewell, Stefan H. Oehlers, Claire M. Mulvey, Philip S. Crosier, Jasminka Godovac-Zimmermann, Stuart L. Bloom, Andrew M. Smith, Anthony W. Segal

**Affiliations:** 1 Division of Medicine, University College London, London, WC1E 6JF, UK; 2 Department of Molecular Medicine and Pathology, University of Auckland, Auckland 1001, New Zealand; 3 Department of Genetics, University of Cambridge, Cambridge, CB2 3EH, UK; 4 Department of Gastroenterology, University College London Hospital, London, NW1 2BU, UK; 5 Microbial Diseases, Eastman Dental Institute, University College London, London, WC1X 8LD, UK

**Keywords:** Crohn's disease, Macrophages, TNFα, *Escherichia coli*, Cytokines

## Abstract

Crohn's disease (CD) is associated with delayed neutrophil recruitment and bacterial clearance at sites of acute inflammation as a result of impaired secretion of proinflammatory cytokines by macrophages. To investigate the impaired cytokine secretion and confirm our previous findings, we performed transcriptomic analysis in macrophages and identified a subgroup of individuals with CD who had low expression of the autophagy receptor optineurin (OPTN). We then clarified the role of OPTN deficiency in: macrophage cytokine secretion; mouse models of bacteria-driven colitis and peritonitis; and zebrafish *Salmonella* infection. OPTN-deficient bone-marrow-derived macrophages (BMDMs) stimulated with heat-killed *Escherichia* *coli* secreted less proinflammatory TNFα and IL6 cytokines despite similar gene transcription, which normalised with lysosomal and autophagy inhibitors, suggesting that TNFα is mis-trafficked to lysosomes via bafilomycin-A-dependent pathways in the absence of OPTN. OPTN-deficient mice were more susceptible to *Citrobacter* colitis and *E. coli* peritonitis, and showed reduced levels of proinflammatory TNFα in serum, diminished neutrophil recruitment to sites of acute inflammation and greater mortality, compared with wild-type mice. *Optn*-knockdown zebrafish infected with *Salmonella* also had higher mortality. OPTN plays a role in acute inflammation and neutrophil recruitment, potentially via defective macrophage proinflammatory cytokine secretion, which suggests that diminished OPTN expression in humans might increase the risk of developing CD.

## Abstract (precis)

**Summary:** Optineurin plays a role in acute inflammation, proinflammatory cytokine secretion and neutrophil recruitment, which suggests that diminished optineurin expression in humans might increase the risk of developing Crohn's disease.

## INTRODUCTION

Crohn's disease (CD) is a chronic relapsing inflammatory disorder, primarily affecting the gastrointestinal tract (Baumgart and Sandborn, 2012). The hallmark of CD is the presence of transmural inflammation with granulomas that commonly involves the terminal ileum.

We previously showed that individuals with CD have defective clearance of bacteria from their tissues, which was associated with inadequate neutrophil recruitment (Segal and Loewi, 1976; Marks et al., 2006; Smith et al., 2009). The impaired secretion of proinflammatory cytokines from macrophages upon bacterial stimulation (Smith et al., 2009; Sewell et al., 2012) could be responsible for this delay in neutrophil recruitment. This abnormal neutrophil response does not occur as a consequence of chronic inflammation: it was not seen in ulcerative colitis (UC) or rheumatoid arthritis (Segal and Loewi, 1976; Marks et al., 2006; Smith et al., 2009). A clear example of the connection between disordered neutrophil function and CD comes from the archetypal neutrophil defect of chronic granulomatous disease (CGD), in which 40% of patients develop bowel disease indistinguishable from CD (Marks et al., 2009). In CGD there is a gross defect of components of the NADPH oxidase that is responsible for the respiratory burst in neutrophils. Mutations in components of this oxidase that are damaging, but not severe enough to cause the oxidase to be seriously compromised, are associated with an increased incidence of early-onset CD (Dhillon et al., 2014).

This led us to propose a ‘three-stage’ model for the pathogenesis of CD. The first stage involves penetration of faecal contents into the bowel wall, which results in the second central causal stage of incomplete bacterial clearance by competent neutrophils. The incomplete clearance of bowel contents from the tissues results in the third stage of a secondary adaptive immune response (Sewell et al., 2009).

The complexity of the cause of CD has been highlighted by recent large-scale genetic studies (Khor et al., 2011). Genome-wide association studies (GWAS) have identified single-nucleotide polymorphisms (SNPs) within 163 susceptibility loci (Franke et al., 2010; Jostins et al., 2012). These loci highlight the importance of genes of the innate immune system that recognise pathogen-associated molecular patterns, such as nucleotide-binding oligomerisation domain-containing 2 (*NOD2*) (Hugot et al., 2001; Ogura et al., 2001), and autophagy, for example autophagy-related 16-like 1 (*ATG16L1*) (Franke et al., 2010; Murthy et al., 2014). However, given the heterogeneity of the CD phenotype and the GWAS limitation of identifying common variants of small effect, it is unsurprising that the GWAS CD-associated variants are calculated to account for only 23% of the total CD heritability (Franke et al., 2010).

In an attempt to identify molecules that might be responsible for disordered macrophage function in CD, we performed a transcriptomic analysis of macrophages from these individuals. Optineurin (OPTN) was identified as a gene with abnormally low expression in approximately 10% of CD patients (Smith et al., 2015). Knockdown of *OPTN* using siRNA resulted in reduced proinflammatory tumour necrosis factor-α (TNF) and interleukin-6 (IL6) secretion upon bacterial stimulation of THP-1 cells, providing evidence that alterations in the expression have a direct impact on the innate immune response to bacterial challenge (Smith et al., 2015).

**TRANSLATIONAL IMPACT**

**Clinical issue**

Crohn's disease (CD) is a chronic inflammatory disorder of the gastrointestinal tract. CD is associated with delayed neutrophil recruitment and bacterial clearance at sites of acute inflammation owing to impaired pro-inflammatory cytokine secretion by macrophages. A subset of individuals with CD have been identified that have reduced macrophage expression of optineurin (OPTN), an autophagy receptor with a role in vesicle trafficking. This study aims to elucidate the role of OPTN deficiency in macrophage cytokine secretion using mouse and zebrafish models of infection.

**Results**

In this study, the authors showed that mouse OPTN-deficient macrophages secrete lower levels of pro-inflammatory cytokines upon *E. coli* stimulation owing to cytokine mis-trafficking to lysosomes via autophagy-dependent pathways. Mice lacking OPTN were significantly more susceptible to *Citrobacter* colitis and *E. coli* peritonitis owing to reduced neutrophil recruitment to sites of acute inflammation and impaired pro-inflammatory cytokine secretion. *Optn*-knockdown zebrafish infected with *Salmonella* also showed higher mortality.

**Implications and future directions**

This study is the first to implicate OPTN in the innate immune response to bacteria in the gut. Reduced OPTN expression is associated with an impaired neutrophil response that increases the risk of developing bacteria-driven colitis and potentially CD. CD due to an innate immunodeficiency resulting from an impaired macrophage and neutrophil response might benefit from the use of lysosomal and autophagy modulators as a new therapeutic strategy in forthcoming clinical trials.

OPTN has been shown to regulate exocytosis of secretory vesicles via interaction with Rab8 and myosin VI at the Golgi complex (Sahlender et al., 2005; Bond et al., 2011), and has a role in post-Golgi protein trafficking and positioning of lysosomes via an interaction with huntingtin (HTT) (del Toro et al., 2009), indicating that dysfunction of OPTN could lead to disordered cytokine secretion. Additionally, phosphorylation of OPTN has been found to promote autophagy of ubiquitylated *Salmonella* (Wild et al., 2011).

*OPTN* gene mutations have previously been associated with primary open angle glaucoma (POAG) (Rezaie et al., 2002), amyotrophic lateral sclerosis (ALS) (Maruyama et al., 2010) and Paget's disease of the bone (Albagha et al., 2010). The most widely studied POAG OPTN mutant is the commonest E50K mutation. Mice overexpressing E50K-OPTN have thinner retinas with loss of retinal ganglion cells (RGCs) (Chi et al., 2010) and impaired post-Golgi trafficking in human retinal pigment epithelium and RGCs. In individuals with ALS, OPTN was found to colocalise with superoxide dismutase 1 (SOD1) and fused in sarcoma (FUS) in inclusion bodies (Maruyama et al., 2010; Ito et al., 2011). Further novel risk variants have been identified in an Italian and Dutch cohort (Del Bo et al., 2011; Tumer et al., 2012), but three other studies did not support the association of OPTN with ALS (Belzil et al., 2011; Millecamps et al., 2011; Solski et al., 2012). The involvement of OPTN in ALS therefore remains to be further elucidated. In 2010, a GWAS into Paget's disease of the bone identified three candidate loci, one of which was mapped to *OPTN* on chromosome 10p13 (Albagha et al., 2010). However, to date the disease-associated variant in OPTN has not been identified, nor have the functional consequences that result in Paget's disease.

In this study, we show that OPTN plays an important role in the inflammatory response and in neutrophil recruitment, which are important in controlling bacterial infection in the bowel (Chew et al., 2014). These studies are the first to identify a role for OPTN in antibacterial responses in the gastrointestinal tract and demonstrate that reduced expression can have a profound effect on the immune response, increasing the likelihood of developing a chronic inflammatory disease such as CD.

## RESULTS

### OPTN deficiency was identified in 10% of CD patients and is associated with inheritance of a minor SNP

To verify our previously reported link between low *OPTN* expression and CD (batch 1) in monocyte-derived macrophages (MDMs) (Smith et al., 2015), we recruited an additional 47 CD patients and 33 healthy controls (HC) (batch 2). Transcriptomic analysis and qPCR verification (data not shown) confirmed our previous findings (Table 1). Low *OPTN* expression was defined as expression below a defined threshold at which the significance (*P*-value) of the standard deviation and fold change of *OPTN* expression in each CD patient when compared to mean expression in HC was *P*<0.005 and 1 on the log_2_ scale, respectively (Smith et al., 2015).

**Table 1.**
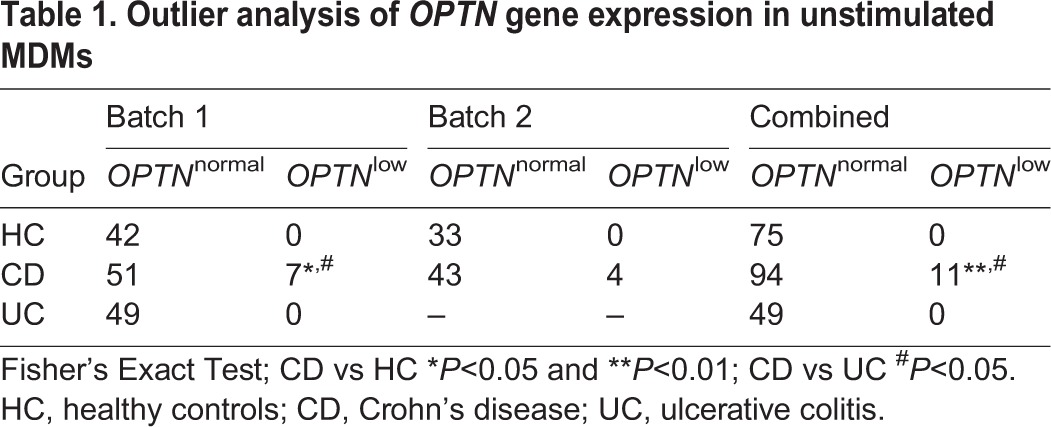
**Outlier analysis of *OPTN* gene expression in unstimulated MDMs**

Of the 105 CD patients in total, 11 (10.5%) were expressing *OPTN* significantly below the range in HC (*n*=75). By contrast, UC patients (*n*=49) demonstrated normal *OPTN* expression (*P*=0.003 and *P*=0.02 comparing individuals with CD with HC and UC, respectively). Having established a link with CD, we looked for any demographic association with low *OPTN* expression. A weak association was found between low *OPTN* expression and male CD patients (*P*=0.011), but otherwise no association was found with age, medication or smoking status (Table 2).

**Table 2.**
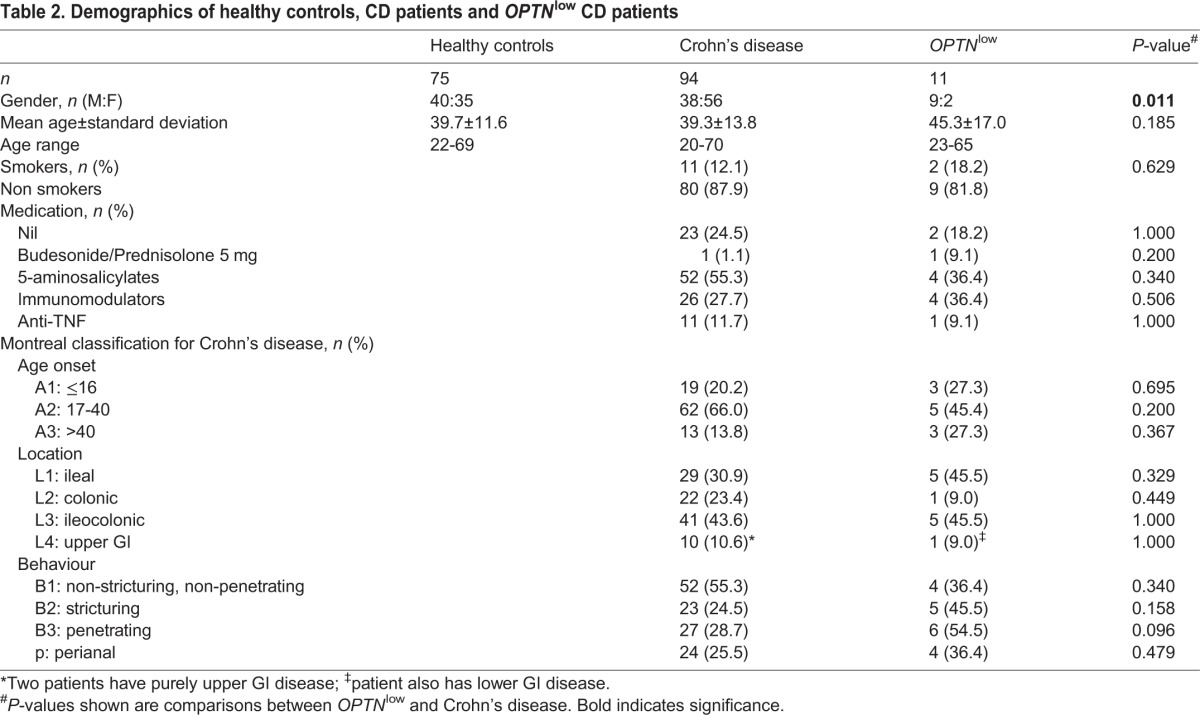
**Demographics of healthy controls, CD patients and *OPTN*^low^ CD patients**

Ten out of eleven of the *OPTN*^low^ CD patients had ileal involvement (Table 3). Ileal biopsies from four *OPTN*^low^ CD patients were stained for OPTN (supplementary material Fig. S1). OPTN staining in lamina propria cells of the ileum is possibly weaker in *OPTN*^low^ patients compared with an *OPTN*^normal^ CD patient (data not shown) and HC small bowel (supplementary material Fig. S1), which suggests that OPTN expression might be reduced in the ileum. We further interrogated a published online dataset (GSE16879) (de Bruyn et al., 2014), which contains ileal biopsy material from 6 controls and 18 CD patients (supplementary material Fig. S2), and found that ileal *OPTN* expression was significantly lower in the CD patients compared with controls (*P*=0.01). To investigate leukocyte OPTN expression, immunoblotting was performed, which demonstrated that OPTN is expressed in monocytes, lymphocytes and MDMs but was undetected in neutrophils (supplementary material Fig. S3).

**Table 3.**
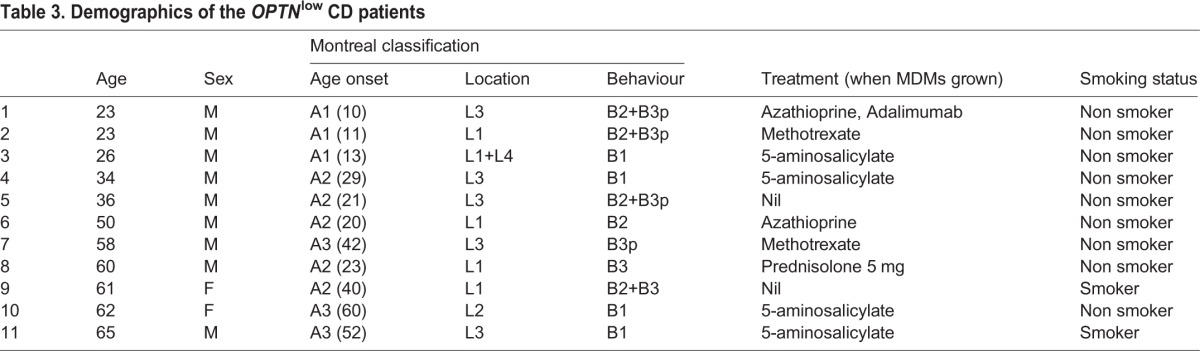
**Demographics of the *OPTN*^low^ CD patients**

### OPTN is upregulated and localises with TNF at the Golgi complex upon exposure to bacteria in human MDMs

**Fig. 1.**
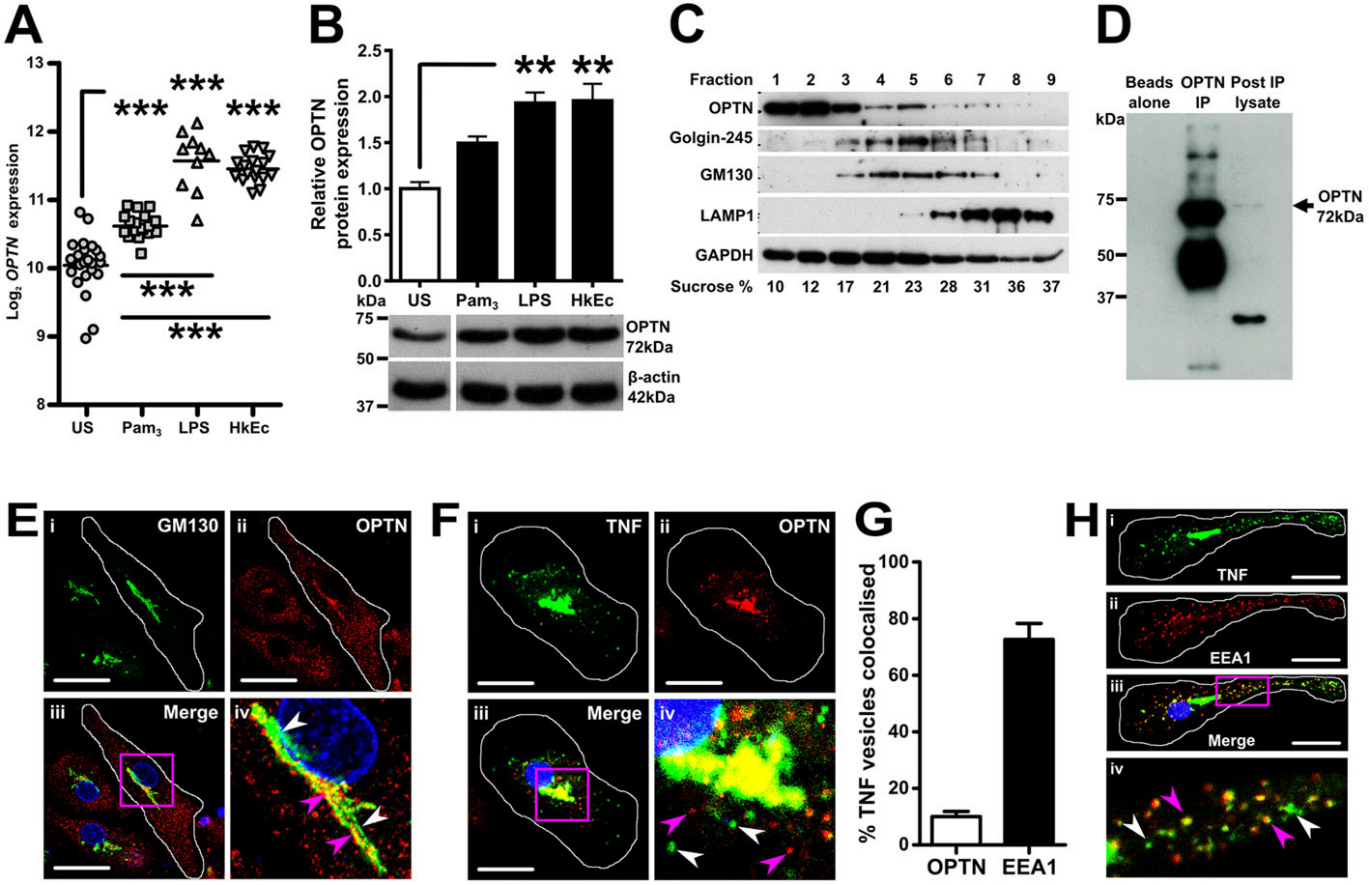
**OPTN is upregulated and colocalises with TNF at the Golgi complex upon bacterial stimulation in macrophages.** (A) *OPTN* expression in MDMs stimulated with Pam_3_ (TLR2), LPS (TLR4) and HkEc compared to unstimulated (US) MDMs (*n*=10-23/group). (B) Immunoblot for OPTN in MDMs after TLR2, TLR4 and HkEc stimulation (*n*=4). (C) Subcellular fractions of HkEc-stimulated THP-1 cells were immunoblotted for OPTN and markers for the Golgi complex (golgin-245 and GM130), lysosomes (LAMP1) and cytosol (GAPDH). (D) Immunoblot for OPTN after immunoprecipitation of OPTN from THP-1 cells. (E) Confocal microscopy in an MDM (white outline) stimulated with HkEc for 4 h were stained for GM130 (i) and OPTN (ii). Single-stained GM130 Golgi complex (white arrowheads) and double-stained OPTN and GM130 (pink arrowheads) are visible (iv). (F) Confocal microscopy in an MDM (white outline) stimulated with HkEc for 4 h were stained for TNF (i) and OPTN (ii), which colocalised within the Golgi complex (iii). Single-stained OPTN vesicles (pink arrowheads) and TNF vesicles (white arrowheads) are visible (iv). (G) Quantification of TNF vesicles that colocalised with OPTN and EEA1 (*n*=8-16 cells/person, 2 persons performed). (H) Confocal microscopy in an MDM (white outline) stimulated with HkEc for 4 h were stained for TNF (i) and early endosome antigen 1 (EEA1) (ii). Double-positive peripheral vesicles (pink arrowheads) and single-positive TNF vesicles (white arrowheads) are shown (iv). Results shown are mean±s.e.m., quantified immunoblots are normalised to actin (**P<0.01 and ***P<0.001; one-way ANOVA and Bonferroni's test for multiple comparisons). All images were taken with a 63× oil-immersion objective. Scale bars: 20 µm.

OPTN was upregulated at the transcriptional and protein levels (Fig. 1A,B) after stimulation with bacteria and microbial ligands in MDMs. Heat-killed *Escherichia* *coli* (HkEc) and lipopolysaccharide (LPS) were the most potent inducers of *OPTN* transcription and resulted in the highest levels of intracellular protein, whereas the Toll-like receptor 2 (TLR2) ligand Pam_3_ was a less potent inducer.

Previous studies have localised OPTN to multiple intracellular locations, including the Golgi complex (Sahlender et al., 2005). Subcellular fractionation was performed on HkEc-stimulated THP-1 cells (Fig. 1C). The majority of OPTN was localised to the cytoplasmic fractions and, to a lesser extent, the Golgi-enriched fraction 5. There was minimal overlap between OPTN- and LAMP1-positive fractions, which suggests that OPTN is not localised to lysosomes.

Immunoprecipitation of OPTN was performed on THP-1 cells, verified by immunoblotting (Fig. 1D) and co-precipitated proteins were identified using shotgun proteomics (supplementary material Table S1). Proteins that co-precipitated with OPTN were subjected to Gene Ontology (GO) analysis, which identified GO-terms associated with intracellular vesicles and the Golgi-network (supplementary material Table S2). The strongest signal associated with the myosin complex was the unconventional myosin 18A (MYO18A), which localises to the trans-Golgi membrane (Dippold et al., 2009).

To confirm the subcellular localisation of OPTN, confocal microscopy was performed. HkEc-stimulated MDM were co-stained for OPTN and the Golgi marker, GM130 (Fig. 1E). There was overlap between OPTN and GM130, replicating the Golgi localisation of OPTN found on subcellular fractionation and immunoprecipitation. OPTN staining was also present within the cytoplasm.

Our previous finding of reduced TNF secretion in OPTN-deficient cells led us to investigate whether TNF and OPTN colocalise in the cell (Smith et al., 2015). Confocal images of HkEc-stimulated MDM demonstrated overlap between OPTN and TNF within the Golgi complex (Fig. 1F, supplementary material Fig. S4). A number of vesicles stained positively for TNF or OPTN, but only 12% of TNF vesicles also co-stained for OPTN (Fig. 1G). Previous studies have shown that TNF is trafficked from the Golgi complex through a Rab11 recycling endosomal pathway in murine macrophages (Manderson et al., 2007). Human MDMs traffic TNF predominantly through the early endosomal pathway (Fig. 1G,H). A total of 70% of TNF-positive vesicles also expressed the endosomal marker early endosome antigen 1 (EEA1), suggesting that human and mouse macrophages might secrete TNF through slightly different endosomal pathways.

### Loss of OPTN in mouse BMDMs results in reduced proinflammatory cytokine secretion on bacterial challenge

We previously showed that OPTN deficiency in human MDMs had no bearing on cytokine gene transcription but diminished the secretion of TNF and IL6 (Sewell et al., 2012; Smith et al., 2015). This pattern of abnormal cytokine secretion was also found in CD patients and associated with defective intracellular protein trafficking (Smith et al., 2009). To investigate the effect of deleting the *OPTN* gene on inflammation and cytokine secretion *in vivo*, we studied *Optn*^−/−^ mice. *Optn*^−/−^ mice were generated on a C57BL/6 background by insertion of a promoter-driven Neo targeting cassette upstream of exon 3 (supplementary material Fig. S5A) (Skarnes et al., 2011), resulting in a 115-bp splice acceptor (SA) insert between exon 2 and 3 on transcription (supplementary material Fig. S5D), which causes a frameshift and multiple predicted premature termination codons on translation.

**Fig. 2.**
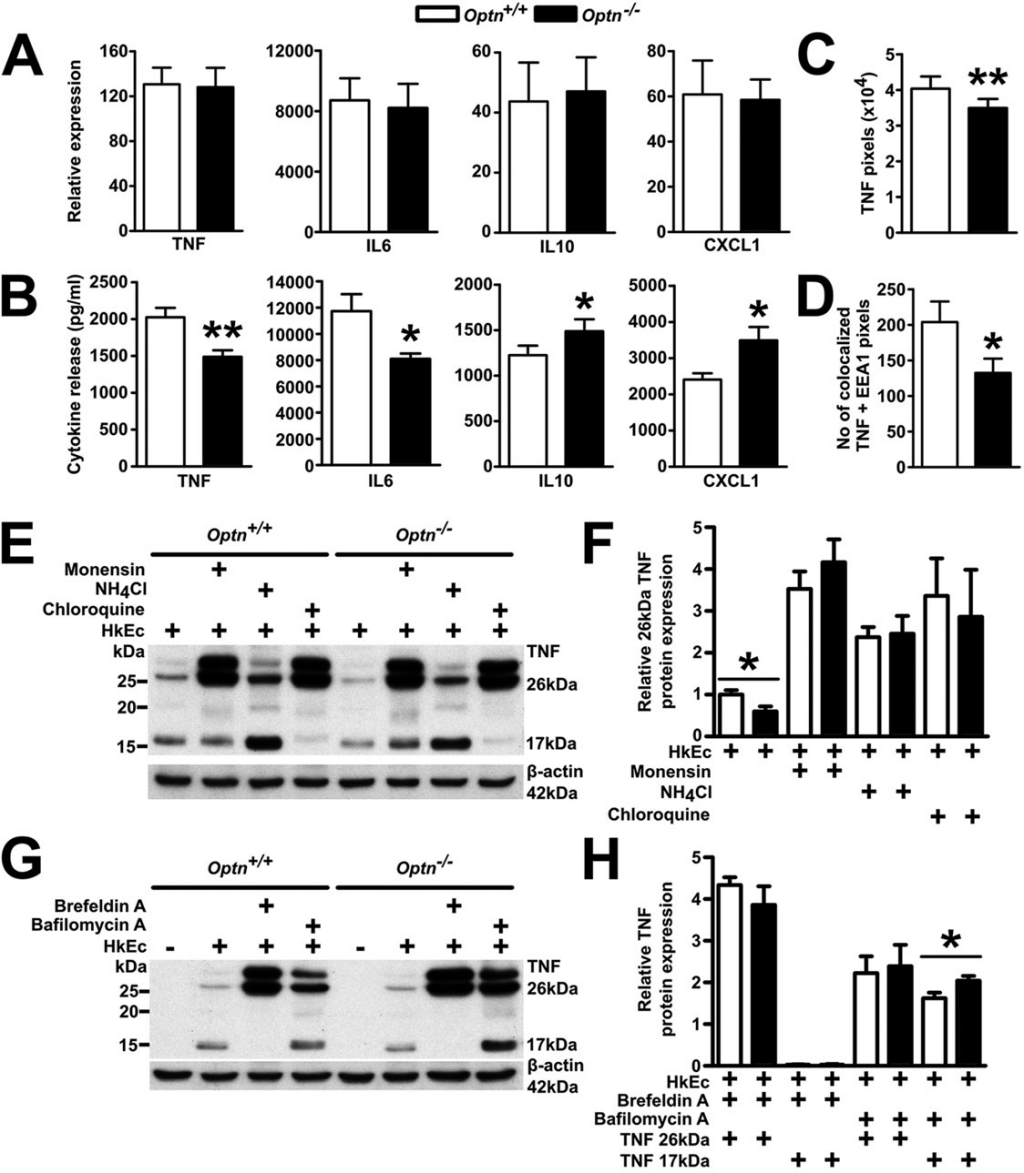
**OPTN-deficient BMDMs secrete lower levels of proinflammatory cytokines on bacterial challenge.** (A) *Tnf*, *Il6*, *Il10* and *Cxcl1* gene expression in *Optn^+/+^* and *Optn^−/−^* BMDMs after stimulation with HkEc was measured with qRT-PCR (*n*=5 mice/group over 5 experiments). (B) TNF, IL6, IL10 and CXCL1 cytokine release in *Optn^+/+^* and *Optn^−/−^* BMDMs after stimulation with HkEc was quantified using a multiplex cytokine plate (*n*=5 mice/group over 5 experiments). (C) Confocal microscopy in BMDMs stimulated with HkEc for 4 h and stained for intracellular TNF were quantified using ImageJ (*n*=177-178 cells/genotype over 2 experiments). (D) Confocal microscopy in BMDMs stimulated with HkEc for 4 h and stained for intracellular TNF and EEA1 were quantified using ImageJ (*n*=62-66 cells/genotype over 2 experiments). (E) Representative TNF immunoblot of whole cell lysates from BMDMs exposed to HkEc in the presence of lysosomal inhibitors. (F) Quantification of immunoblots for TNF showed significantly less intracellular 26-kDa precursor TNF in the BMDMs from *Optn^−/−^* mice, which is normalised to wild-type levels on addition of lysosomal inhibitors monensin, NH_4_Cl or chloroquine (*n*=3-8 mice/genotype over 3-5 experiments). (G) Representative TNF immunoblot of whole cell lysates from naïve and HkEc-stimulated BMDMs in the presence of brefeldin A or bafilomycin A. (H) Quantification of immunoblots for TNF in the presence of brefeldin A shows undetectable levels of the 17-kDa secreted form of TNF, whereas bafilomycin A results in significantly higher levels of the 17-kDa secreted TNF in *Optn^−/−^* BMDMs (*n*=3-8 mice/genotype over 3-5 experiments). Results shown are mean±s.e.m., all immunoblots are normalised to actin (*P<0.05, and **P<0.01; two-tailed, unpaired *t*-test).

Whereas cytokine gene transcription in HkEc-stimulated bone-marrow-derived macrophages (BMDMs) were upregulated to similar levels in both *Optn^+/+^* and *Optn^−/−^* mice (Fig. 2A), cytokine secretion differed significantly (Fig. 2B), with reduced TNF (*P*=0.006) and IL6 (*P*=0.023) and elevated IL10 (*P*=0.028) and chemokine (C-X-C motif) ligand 1 (CXCL1) (*P*=0.024) in *Optn^−/−^* BMDMs. This cytokine secretion defect occurred despite normal *E. coli* phagocytosis and killing, autophagy induction, and the absence of a difference in endoplasmic reticulum stress in *Optn^−/−^* BMDM (supplementary material Figs S6-S8).

TNF trafficking via the endosomal pathway in BMDMs was investigated by confocal microscopy, which demonstrated reduced levels of intracellular TNF (Fig. 2C, supplementary material Fig. S9) and significantly less colocalisation of TNF-positive vesicles with EEA1 in *Optn^−/−^* BMDMs (Fig. 2D). Immunoblotting of TNF further confirmed the reduced intracellular TNF in HkEc-stimulated *Optn^−/−^* BMDMs (Fig. 2E,F).

To investigate whether the reduced intracellular TNF was due to defective protein translation in *Optn^−/−^* BMDMs, we included brefeldin A, a potent inhibitor of protein transport from the endoplasmic reticulum to the Golgi. Intracellular TNF has previously been shown to exist as a membrane-bound 26-kDa precursor and a 17-kDa secreted form (Shurety et al., 2000). Stimulation of BMDMs with HkEc in the presence of brefeldin A resulted in elevation of the 26-kDa precursor TNF and loss of the 17-kDa secreted form to similar levels in both *Optn^+/+^* and *Optn^−/−^* BMDMs (Fig. 2G,H), demonstrating that TNF is translated normally in the absence of OPTN.

By contrast, the inclusion of bafilomycin A, an inhibitor of vacuolar type H^+^-ATPase that blocks fusion between autophagosomes and lysosomes, resulted in higher intracellular levels of the 17-kDa secreted TNF form (*P*=0.041) in the *Optn*^−/−^ compared with *Optn^+/+^* BMDMs, with no difference in the 26-kDa precursor TNF form, indicating that, in the absence of OPTN, a greater proportion of the secreted form of TNF is trafficked through a bafilomycin-A-sensitive intracellular compartment in BMDMs.

Inhibition of lysosomal proteases has been shown to increase intracellular cytokine levels in HkEc-stimulated human MDMs and to correct the deficient levels found in cells from CD patients (Smith et al., 2009). Therefore, we investigated the role of the lysosome in intracellular TNF trafficking with the inclusion of monensin, chloroquine and NH_4_Cl. In HkEc-stimulated BMDMs, all three inhibitors of lysosomal degradation increased intracellular levels of TNF to equal levels in *Optn^−/−^* and *Optn^+/+^* cells when compared with HkEc stimulation alone (Fig. 2E,F), indicating that a greater proportion of intracellular TNF is directed to lysosomal degradation in the absence of OPTN.

### OPTN confers protection against bacterial infection in mice and zebrafish

**Fig. 3.**
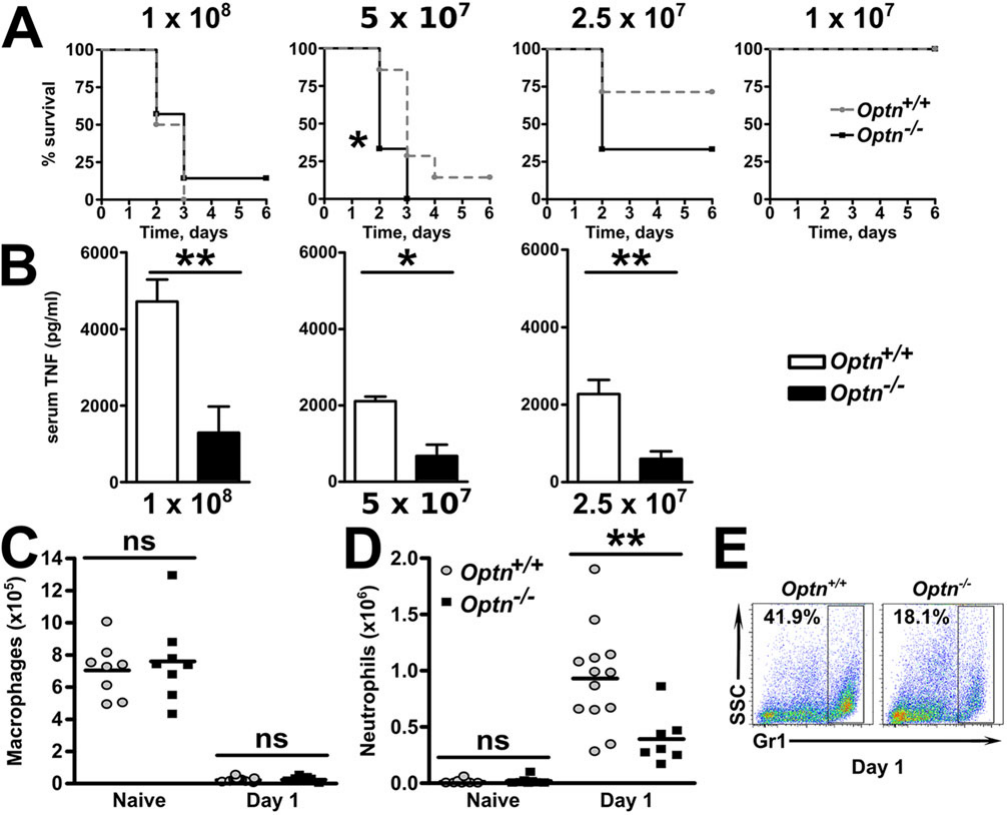
**OPTN is protective against *in vivo* bacterial infection.** (A) *Optn^+/+^* and *Optn^−/−^* mice were injected with different quantities of live *E. coli* into their peritoneum (*n*=4-12 mice/genotype over 3 experiments). (B) Mice were tail bled at day 2 to measure serum TNF levels. (C,D) Peritoneal washouts were performed on naïve *Optn^+/+^* and *Optn^−/−^* mice and at day 1 after *E. coli* inoculation into the peritoneum for flow cytometry of (C) CD11b^+^ F4/80^+^ macrophages and (D) Gr1^+^ neutrophils (*n*=7-13 mice/genotype over 2 experiments). (E) Representative flow cytometry of Gr1^+^ neutrophils in *Optn^+/+^* and *Optn^−/−^* mice at day 1. Results shown are mean±s.e.m. (*P<0.05 and **P<0.01; two-tailed, unpaired *t*-test).

To determine the importance of OPTN in host antibacterial response, we employed models of infection in two evolutionarily divergent species. When peritonitis was induced in mice with live *E. coli*, there was a dose-dependent increase in the mortality of both *Optn^+/+^* and *Optn^−/−^* mice (Fig. 3A), but the *Optn^−/−^* mice seemed to be slightly more susceptible to *E. coli* inoculation. Compatible with the observed reduction of the release of TNF from cultured macrophages, the levels of TNF in the circulation were lower in *Optn^−/−^* mice (Fig. 3B).

The numbers of macrophages and neutrophils in the naïve peritoneum of *Optn^+/+^* and *Optn^−/−^* mice were not different (Fig. 3C,D). 24 h after induction of *E. coli* peritonitis, there was a significant reduction in the number of macrophages in the peritoneal washout in both *Optn^+/+^* and *Optn^−/−^* mice (Fig. 3C), with no significant difference between them. By contrast, neutrophil numbers were significantly increased (Fig. 3D) but the elevation in *Optn^+/+^* animals was significantly greater than in *Optn^−/−^* mice (*P*=0.007) (Fig. 3D,E).

To determine the importance of OPTN in antibacterial response across species, we performed preliminary experiments in a zebrafish infection model involving *Salmonella enterica*. As previously shown in human MDMs (Fig. 1A,B) and mouse macrophages (supplementary material Fig. S5E), whole zebrafish *optn* expression was elevated after bacterial stimulation with *Salmonella* via both an oral (immersion) infection and direct injection of bacteria into embryos (supplementary material Fig. S10). To study the consequence of Optn deficiency in zebrafish antibacterial responses, *optn-*specific morpholinos (MOs) were used. *optn* knockdown resulted in an increased susceptibility to *Salmonella* infection compared with wild-type fish and those treated with control MO (supplementary material Fig. S11).

### OPTN deficiency results in an exaggerated *Citrobacter*-induced colitis

**Fig. 4.**
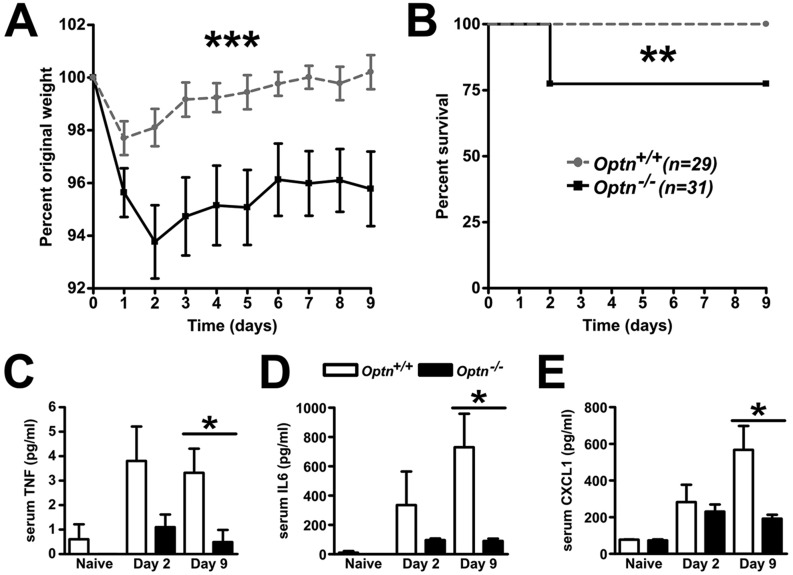
***Citrobacter* colitis results in greater mortality and lower levels of serum pro-inflammatory cytokines in OPTN-deficient mice.** (A,B) *Optn^+/+^* (grey) and *Optn^−/−^* (black) mice were gavaged with live *Citrobacter rodentium* and followed for 9 days to assess (A) weight loss and (B) mortality (*n*=29-31 mice/genotype over 5 experiments). (C) Serum TNF, (D) IL6 and (E) CXCL1 were measured in naïve mice, day 2 and day 9 after *Citrobacter* inoculation, in tail bleed and cardiac puncture serum (*n*=5 mice/group). Results shown are mean±s.e.m. (*P<0.05, **P<0.01 and ***P<0.001; two-tailed, unpaired *t*-test and logrank test).

To investigate the antibacterial role of OPTN in the bowel, a *Citrobacter*-induced colitis model was used. Following *Citrobacter* inoculation, the *Optn^−/−^* mice demonstrated increased susceptibility in the first 2 days with significantly greater weight loss (Fig. 4A) and mortality (Fig. 4B). This increased susceptibility was not due to a *Citrobacter* bacteraemia (data not shown) or differences in faecal *Citrobacter* levels (supplementary material Fig. S12).

*Citrobacter* inoculation resulted in increased serum levels of TNF, IL6 and CXCL1 in *Optn^+/+^* and *Optn^−/−^* mice at day 2 compared with naïve animals (Fig. 4C-E). In the *Optn^+/+^* mice, there was a further increase in the levels of IL6 and CXCL1 at day 9, whereas the level of TNF remained similar to that on day 2. *Optn^−/−^* mice released equivalent levels of CXCL1 at day 2 but demonstrated a less pronounced increase in TNF and IL6 in comparison to wild-type animals. By day 9 post *Citrobacter* inoculation, TNF, IL6 and CXCL1 were all significantly lower in *Optn^−/−^* compared to *Optn^+/+^* mice. *TNF* mRNA expression in the bowel at day 2 and day 9 was no different from wild-type mice (supplementary material Fig. S13).

**Fig. 5.**
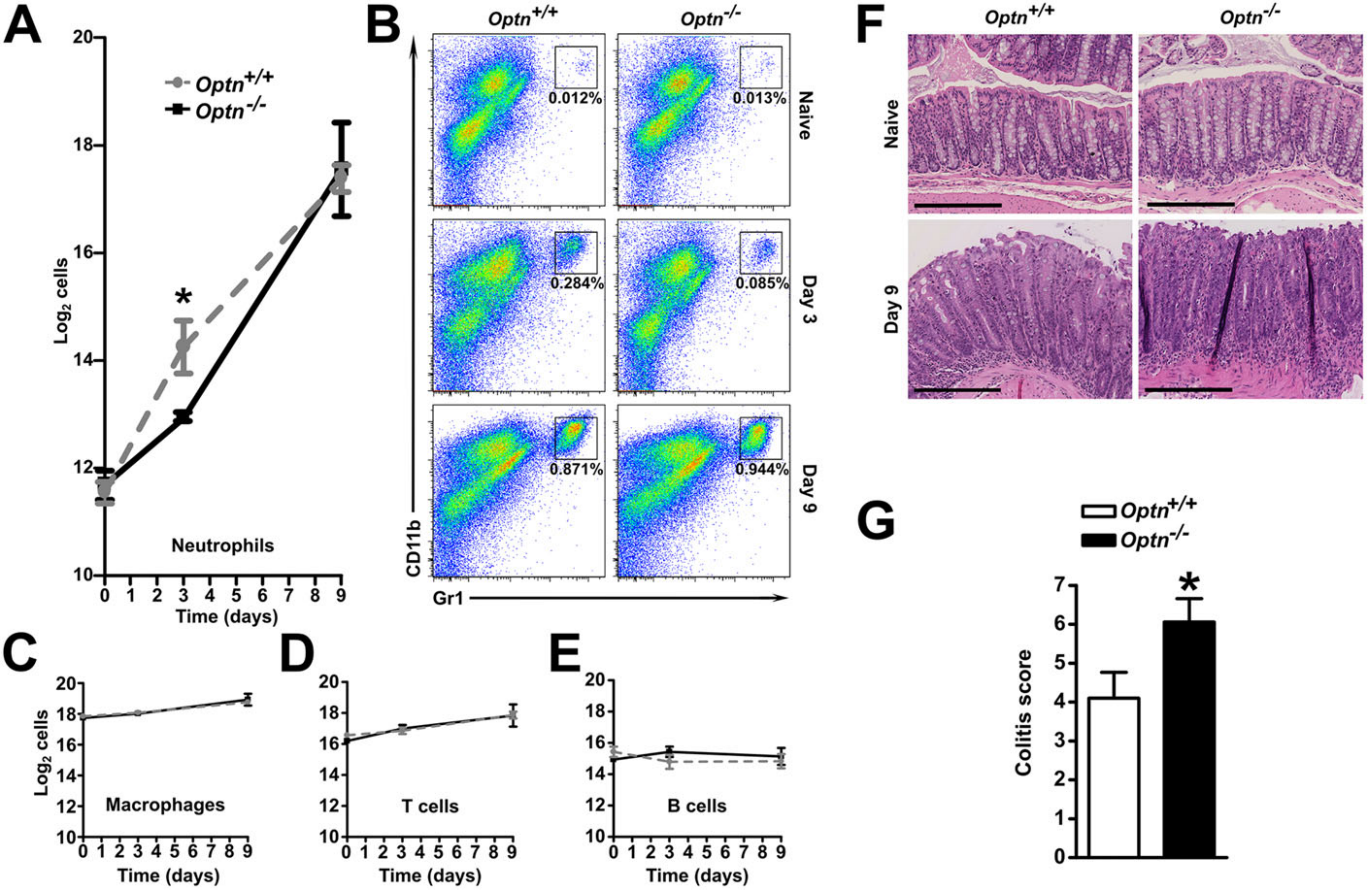
**OPTN-deficient mice recruit fewer neutrophils during the early acute phase of *Citrobacter* infection, resulting in a more severe colitis.** (A) Large bowel from naïve mice, day 3 and day 9 after *Citrobacter* inoculation, were digested and flow cytometry for CD11b^+^ Gr1^+^ neutrophils was performed (*n*=10-11 mice/genotype over 3 experiments). (B) Representative flow cytometry plots for CD11b^+^ Gr1^+^ neutrophils in naïve mice, day 3 and day 9 after *Citrobacter* inoculation, are shown with percentage of neutrophils. (C) Flow cytometry for CD11b^+^ F4/80^+^ macrophages, (D) CD3^+^ T cells and (E) CD19^+^ B cells in large bowels from naïve mice, day 3 and day 9. (F) Representative image of haematoxylin and eosin (H&E)-stained naïve and day 9 after *Citrobacter* inoculation large bowel tissue (20× magnification, scale bar: 200 µm). (G) Blinded colitis scoring of H&E-stained large bowel sections at day 9 in *Optn^+/+^* and *Optn^−/−^* mice (*n*=17-20 mice/genotype, over 2 experiments). Results shown are mean±s.e.m. (*P<0.05; two-tailed, unpaired *t*-test).

To investigate the cellular composition of the inflammatory milieu, we performed flow cytometry of cells recovered from whole colonic tissue. Naïve tissue contained equivalent numbers of macrophages, B cells, T cells and neutrophils in both *Optn^+/+^* and *Optn^−/−^* mice (Fig. 5A-E). At 3 days after infection with *Citrobacter*, there was a significant increase in neutrophil numbers within the *Optn^+/+^* and *Optn*^−/−^ colons (Fig. 5A). This early recruitment of neutrophils was significantly attenuated in *Optn*^−/−^ mice (*P*=0.024), which equalised by day 9 (Fig. 5A,B). There were no changes in the numbers of macrophages or T cells at day 3 but, by day 9, both populations had significantly increased (Fig. 5C,D). B cells were unchanged throughout the inflammatory episode (Fig. 5E).

Uninfected colons of *Optn^+/+^* and *Optn*^−/−^ mice were histologically indistinguishable, with no evidence of inflammation (Fig. 5F). At 9 days after *Citrobacter* infection, both groups of mice developed colitis (Fig. 5F), but the colitis score was significantly higher in the *Optn*^−/−^ mice (Fig. 5G).

By contrast, *Optn^−/−^* mice exhibited a dextran sodium sulphate (DSS)-induced colitis phenotype indistinguishable from wild-type animals, with similar weight loss, mortality and levels of serum TNF (supplementary material Fig. S14).

## DISCUSSION

The present results demonstrate a role for OPTN in bowel inflammation *in vivo*. Bacterial exposure upregulates OPTN expression, whereas OPTN deficiency results in impaired proinflammatory cytokine secretion, diminished recruitment of neutrophils into acutely inflamed tissue, an exaggerated colitis and increased susceptibility to bacterial infection. The phenotype of diminished neutrophil recruitment identified in OPTN-deficient mice mirrors our published findings in CD patients (Smith et al., 2009), reinforcing the suggestion that reduced expression of OPTN could play an important role in the development of bacteria-driven colitis in humans.

The identification of reduced *OPTN* expression in approximately a tenth of our CD patients provided evidence that it might play a role in the cytokine secretion defect observed in CD, underpinned by its established role in vesicle trafficking and secretion (Sahlender et al., 2005). None of the commonly published glaucoma, ALS and Paget's variants was identified in these *OPTN*^low^ CD patients. The low *OPTN* expression in human macrophages has previously been associated with the inheritance of a minor allele (rs12415716) in an intronic region downstream of the last exon of the *OPTN* gene (Smith et al., 2015), which tags a region spanning exons 7-16. The expression of *OPTN* in MDMs influenced by inheritance of this minor allele was shown to be exaggerated in individuals with CD; the cause of this difference is still unknown.

We previously demonstrated that proinflammatory cytokine secretion was reduced upon depletion of OPTN in the monocytic cell line THP-1 (Smith et al., 2015). We have expanded these initial findings and identified a major *in vivo* role for OPTN in the inflammatory response in the bowel. The loss of OPTN in knockout mice and diminished levels in CD patients did not result in complete loss of cytokine secretion. However, partial impairment of the secretion of proinflammatory cytokines might be pathologically relevant when the host is exposed to a large quantity of bacteria requiring an immediate, robust inflammatory response. This type of defect is highly relevant in the case of CD, for which we have previously demonstrated a dose-dependent immune deficiency in these individuals (Farthing, 2004; Smith et al., 2009). A breach in the mucosal wall could result in the exposure of the underlying tissue to an enormous bacterial challenge, which needs rapid containment and clearance. Reduced OPTN expression, or inheritance of NOD2, ATG16L1 variants or congenital monogenic innate immunodeficiencies will impact directly on the efficiency of this response, failure of which could result in inadequate clearance of foreign material and the development of chronic inflammation.

OPTN has been shown to localise to the Golgi complex in multiple cell types and play an important role in vesicle trafficking through the formation of complexes with binding partners such as Rab8, HTT and myosin VI. The role and composition of the OPTN-containing complex in vesicle trafficking is only partially elucidated. Previous studies have provided evidence of a role in protein sorting within the Golgi complex and trafficking to the plasma membrane (Sahlender et al., 2005). Replacement of OPTN's binding partner HTT with a 111Q polyglutamine mutant resulted in reduced localisation of OPTN to the Golgi complex and impaired post-Golgi trafficking in striatal cells (del Toro et al., 2009). Depletion of *OPTN* with siRNA in HeLa cells also resulted in dramatically reduced exocytosis. However, these studies did not look at the role of OPTN in the context of bacterial or TLR stimulation that is relevant in the context of CD. Recently, a mouse in which wild-type OPTN was replaced by the polyubiquitin-binding-defective OPTN mutant demonstrated an abrogated response to LPS in BMDMs due to impaired activation of TANK-binding kinase 1 (Gleason et al., 2011). This was associated with defective interferon-β release, but TNF and IL6 secretion was described as normal (Munitic et al., 2013). This is in contrast to our findings of defective cytokine secretion, which indicates that TNF and IL6 secretion is partially dependent on the expression of OPTN but independent of its ability to bind ubiquitin in BMDMs.

**Fig. 6.**
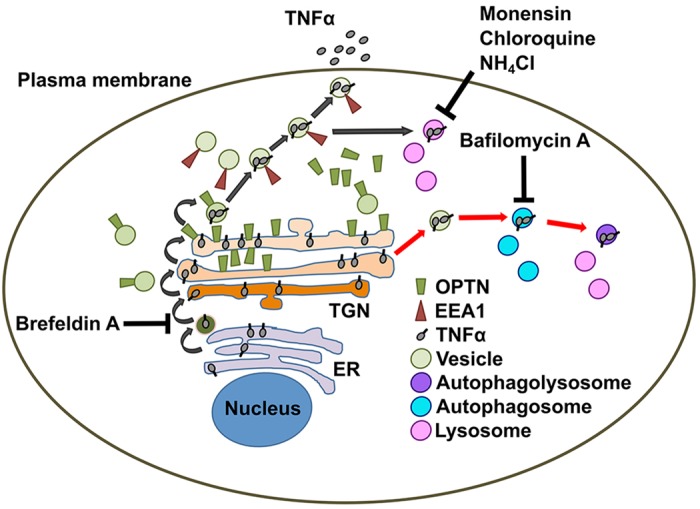
**Schematic diagram of TNF trafficking in murine macrophages.** TNF is translated in the endoplasmic reticulum (ER), transferred to the *trans*-Golgi network (TGN) for post-translational modification before packaging into vesicles, and is trafficked via early endosome antigen 1 (EEA1)-positive vesicles to the plasma membrane for secretion (black arrows). In the absence of OPTN, a greater proportion of TNF is trafficked and degraded via bafilomycin-A-dependent pathways (red arrows). Monensin, chloroquine and NH_4_Cl inhibit lysosomal function, whereas brefeldin A blocks ER-to-Golgi transfer.

Cytokine trafficking in macrophages is complex and only partially understood. Previous work in murine macrophages has shown that TNF and IL6 share a common pathway of secretion from the trans-Golgi network to endosomes (Murray et al., 2005; Manderson et al., 2007). OPTN only minimally colocalises to the endosomal compartment (Mankouri et al., 2010). BMDMs that lack OPTN demonstrate a reduced overlap between TNF and the endosomal compartment. These findings would suggest that the role of OPTN in the defective release of TNF and IL6 in BMDMs lies in its role in sorting proteins for transport at the Golgi complex. Inclusion of a range of protein trafficking inhibitors results in increasing the level of TNF within the cells. The reduction in secreted protein coupled with the generation of equivalent levels of intracellular protein after bacterial stimulation provides evidence to support our previous findings in CD patients of cytokine mis-trafficking. Inclusion of bafilomycin A, an inhibitor of vacuolar H^+^-ATPases, results in a greater intracellular build-up of the 17-kDa secreted form of TNF in *Optn^−/−^* BMDMs than wild-types. These findings suggest that, in the absence of OPTN, a greater proportion of TNF is trafficked through a V-ATPase-dependent compartment and degraded instead of being released at the plasma membrane (Fig. 6).

Reduced TNF and IL6 release was shown to coincide with defective neutrophil recruitment after bacteria-induced inflammation in the bowel and peritoneum of *Optn^−/−^* mice. In both inflammatory models, an increased systemic response was recorded that contributed to an increased colitis and greater mortality. Alterations in cytokine levels can have profound effects on inflammatory episodes. Lamina propria macrophages are replenished from peripheral blood monocytes (Ginhoux and Jung, 2014) and remain one of the key regulators of the immune response (Medzhitov, 2008; Smith et al., 2009) in the large bowel. TLR stimulation results in the induction of local cytokine production (Marks et al., 2006), which will contribute to the neutrophil recruitment seen during *Citrobacter*-induced inflammation. It is therefore plausible that, in OPTN-deficient animals, the loss in cytokine secretion from lamina propria macrophages results in diminished recruitment of neutrophils. It is also possible that other OPTN-deficient cells in the bowel, such as intestinal epithelial cells, might, alone or in conjunction with macrophages, contribute to defective neutrophil recruitment. However, the attenuated neutrophil recruitment and reduced cytokine secretion in the bowel is also seen in the peritoneum, suggesting that a similar defect is active in both sites. Macrophages have been shown to play a central role in gut *Citrobacter* infection (Schreiber et al., 2013) and peritoneal *E. coli* infection (Dunn et al., 1985). This, coupled with the fact that BMDMs from OPTN-deficient animals release significantly reduced levels of TNF and IL6 after *Citrobacter* and *E. coli* stimulation, is highly suggestive that macrophages are responsible for the observed phenotype.

Other studies have demonstrated an elevation in inflammation resulting from a deficiency in TNF expression (Marino et al., 1997). *TNF*-knockout mice are more susceptible to bacterial infection and generate a more severe colitis than control mice (Naito et al., 2003; Xu et al., 2007). These findings provide evidence that TNF has a protective role in the acute phase of an inflammatory response and the release of suboptimal levels could result in the development of chronicity. TNF and IL6 have also been shown to be protective in *Citrobacter* colitis (Goncalves et al., 2001), and suppression of TNF and IL6 expression inhibits intestinal immunity to *Citrobacter* infection (Yan et al., 2012).

The difference in response to DSS and *Citrobacter* might highlight the role of OPTN in bacterial handling, in contrast to simply responding to chemical-induced tissue damage and inflammation. The precise mechanism of DSS-induced colitis is still not fully understood but is widely accepted to result from direct damage to the colonic epithelial monolayer by DSS, allowing the penetration of proinflammatory intestinal contents into the underlying tissue (Perse and Cerar, 2012). The major site of inflammation in DSS-induced colitis is the distal colon, more akin to UC (Chassaing et al., 2014), resulting in the accumulation of innate and adaptive cells in the acute colitis phase (Hall et al., 2011). In contrast to DSS, infection by *Citrobacter* predominantly infects the proximal colon, resulting in the most pronounced inflammation occurring here (Collins et al., 2014). The alternative sites of inflammation, inflammatory stimulus and induction of different inflammatory cell types might provide some explanation for the different response to DSS and *Citrobacter* observed in the OPTN-deficient mice. These findings are not unique, because mice deficient in NOD2, which is strongly associated with CD, are similarly susceptible to a *Citrobacter* (Kim et al., 2011) and *Salmonella* (Claes et al., 2014) colitis but not to DSS (Kobayashi et al., 2005). NOD2 deficiency results in impaired cytokine secretion and diminished phagocyte recruitment during *Citrobacter* infection compared to wild-type animals, a phenotype that mirrors the one we have identified in the *Optn^−/−^* mice (Kim et al., 2011). These findings reinforce the theory that an impaired innate immune response to bacterial infection can lead to the development of chronic bowel inflammation.

Preliminary data in zebrafish suggests that OPTN is protective against bacterial infection, replicating the findings in mice. These results suggest an evolutionarily conserved role for OPTN in immunity against bacterial infection. This preservation in function is maintained in zebrafish despite only sharing 41% homology with mice and 46% with human *OPTN* (Bosenko et al., 2004). The fact that the antibacterial function of OPTN has been conserved between fish and mammals suggests that this is a potentially major evolutionary advantageous property. The loss of this immune mechanism results in an increased bacterial susceptibility and the development of chronic tissue inflammation. However, the *Salmonella* susceptibility studies need to be interpreted with caution because off-target events can occur using MO technology (Bedell et al., 2011) and further, more refined, studies will be needed to characterise the role of *optn* in zebrafish innate immunity.

In conclusion, we have identified a novel role for OPTN in proinflammatory cytokine secretion, neutrophil recruitment and bowel inflammation. The phenotype of diminished neutrophil recruitment demonstrated in OPTN deficiency mirrors our published findings in CD patients and suggests that low *OPTN* expression might contribute to an attenuated antibacterial response and the development of CD.

## MATERIALS AND METHODS

### Patients and healthy controls

This study was approved by the Joint University College London (UCL)/UCL Hospitals Ethics Committee and the NHS London-Surrey Borders Ethics Committee. CD patients were recruited from the UCLH IBD clinic with matched healthy controls (HC) from UCL staff and students. Patients had definitive diagnoses of CD made using standard diagnostic criteria and the Montreal classification for CD. CD patients who were between 18 and 75 years of age, had a diagnosis made more than 1 year previously that was histologically and clinically consistent, had quiescent disease on the Harvey-Bradshaw index, were on stable treatment for the past 3 months, did not have hepatitis B, C or HIV, and were not pregnant were recruited. Age-matched HCs with no personal or family history of CD and were not on immunosuppressants were also recruited. Written informed consent was obtained from all participants.

### *OPTN* sequencing and macrophage expression microarray

Genomic DNA was extracted from peripheral blood using the QIAamp DNA blood Mini Kit (Qiagen, Crawley, UK) and total RNA was harvested using the RNeasy^®^ Mini Kit with RNase-free DNase treatment (Qiagen) and analysed as previously published (Smith et al., 2015).

### Quantitative reverse transcription-PCR (qRT-PCR)

Total RNA from BMDMs in Buffer RLT (Qiagen) and TissueLyser LT (Qiagen) homogenised large bowel in RNA*later* (Qiagen) were harvested using the RNeasy^®^ Mini Kit (Qiagen). Total RNA was converted to complementary DNA (cDNA) using the QuantiTect^®^ Reverse Transcription Kit (Qiagen). qRT-PCR of *Tnf*, *Il6*, *Il10* and *Cxcl1* was performed using the QuantiFast SYBR^®^ Green PCR Kit (Qiagen), in duplicate on a Mastercycler^®^ ep *realplex* (Eppendorf, Stevenage, UK) with primers created using Primer3 (supplementary material Table S3). Normalised mean gene expression values±s.d. were determined from duplicate cycle threshold (Ct) values for each gene and the housekeeping gene peptidylprolyl isomerase A (*Ppia*) and determined by the 2−ΔΔCt method (Livak and Schmittgen, 2001).

### *Optn^+/+^* and *Optn^‒/‒^* mice

Animal studies were performed in accordance with the United Kingdom Animals (Scientific Procedures) Act 1986 and European Directive 2010/63/EU on the protection of animals used for scientific purposes. C57BL/6NTac-Optn^tm1a(EUCOMM)Wtsi^ mice were generated by the Wellcome Trust Sanger Institute, Cambridge as previously described (supplementary material Fig. S5) (Skarnes et al., 2011).

To exclude off-target effects of the Neo cassette, qRT-PCR of *Mcm10* and *Ccdc3*, which flank *Optn* on chromosome 2, was performed in BMDMs, which demonstrated no difference in expression between *Optn*^+/+^ and *Optn*^–/–^ cells (supplementary material Fig. S5B). Genotyping of *Optn*^+/+^ and *Optn*^–/–^ mice was performed using ear clip genomic DNA (supplementary material Fig. S5C). The *Optn* Neo cassette resulted in a 115 base-pair splice acceptor (SA) insert that causes a frameshift and multiple premature termination codons (supplementary material Fig. S5D), confirmed by the lack of the OPTN protein in *Optn*^–/–^ thioglycollate-induced peritoneal macrophages (TiPM) by immunoblot (supplementary material Fig. S5E).

TiPM were stimulated with muramyl dipeptide (MDP), Pam_3_ and HkEc for 24 hours (supplementary material Fig. S5E). As has previously been shown with human MDM (Fig. 2B), HkEc was the most potent inducer of OPTN compared to Pam_3_ and MDP (supplementary material Fig. S5E). *Optn*^–/–^ mice did not develop a spontaneous colitis and were no different from *Optn*^+/+^ mice in terms of weight gain, litter size, gross organ histology and peripheral blood CD3^+^ T cell, CD19^+^ B cell, Ly6C^+^ monocytes or Gr1^+^ neutrophils (data not shown).

### Cell culture and stimulation

Peripheral blood monocytes were isolated using Lymphoprep™ (Axis-Shield, Stockport, UK) and cultured for 5 days to obtain adherent monocyte-derived macrophages (MDMs) as previously described (Rahman et al., 2010). MDMs were plated overnight in X-VIVO™ 15 medium (Lonza, Tewkesbury, UK) at 10^6^ cells on 35 mm Nunclon™ Δ coated tissue culture plates (Nunc, Loughborough, UK) for total RNA, at 2.5×10^5^ cells/well in FALCON^®^ 24-well tissue culture plates for immunoblotting or 10^5^ cells/well in FALCON^®^ 96-well tissue culture plates for cytokine assays, then stimulated with 1 µg/ml MDP (Sigma, Dorset, UK), 4 µg/ml Pam_3_CSK_4_ (Alexis Biochemicals, Exeter, UK), 200 ng/ml lipopolysaccharide (LPS) (Alexis Biochemicals) or heat-killed *E. coli* (HkEc) NCTC 10418 at a multiplicity of infection (MOI) of 20. Human THP-1 acute monocytic leukaemia cells were cultured in RPMI-1640, GlutaMAX™ Supplement (Gibco, Paisley, UK) containing 10% FBS (Sigma), 100 U/ml penicillin, 100 µg/ml streptomycin (Gibco), 20 mM HEPES (Sigma) and 20 µM β-mercaptoethanol (Gibco), plated and stimulated as above. For bone-marrow-derived macrophages (BMDMs), bone marrow cells were harvested from *Optn^+/+^* and *Optn^−/−^* mice aged 9 to 12 weeks and treated with red-blood-cell lysis buffer (Sigma). The remaining cells were cultured in Dulbecco's Modified Eagle Medium (DMEM) (Gibco) containing 1 g/l D-glucose, 4 mM L-glutamine, 25 mM HEPES, 1 mM pyruvate, 10% FBS (Sigma), 100 U/ml penicillin, 100 µg/ml streptomycin (Gibco) and 20 ng/ml M-CSF (Peprotech, London, UK) on 92 mm Nunclon™ Δ coated tissue culture plates (Nunc) for 5 days. BMDMs were plated overnight in DMEM then stimulated as above. To obtain thioglycollate-induced peritoneal macrophages, mice were injected with 1 ml of sterile aged 3% thioglycollate broth (Merck, Nottingham, UK) intraperitoneally. After 5 days, cells were harvested in cell dissociation buffer (Gibco), plated in RPMI-1640, GlutaMAX™ Supplement (Gibco) containing 10% FBS (Sigma), 100 U/ml penicillin, 100 µg/ml streptomycin (Gibco), 20 mM HEPES (Sigma) and stimulated as above. Cytokine levels in supernatants were measured using the Mouse Proinflammatory Ultrasensitive plate (Meso Scale Discovery, Rockville, MD, USA).

### Subcellular fractionation

Sucrose gradients were prepared by layering eight 5% step dilutions of a 50% sucrose solution containing 1 mM EDTA pH 7.4 and 5 U/ml heparin, which was left overnight to equilibrate at 4°C. 2×10^8^ THP-1 cells were stimulated with HkEc at a MOI of 20 for 24 h then dounced and sonicated 3×5 s twice in 10% sucrose containing 1 mM EDTA pH 7.4, 5 U/ml heparin and protease inhibitors on ice. Cells were confirmed to be lysed on light microscopy and centrifuged at 750 ***g*** for 10 min at 4°C. The post-nuclear supernatant was layered onto the sucrose gradient and ultracentrifuged in a TST 41.14 Kontron swing-bucket rotor at 220,000 ***g*** for 3 h at 4°C on a Beckman Optima™ LE-80K Ultracentrifuge (Beckman, High Wycombe, UK). The subcellular fractions were removed in 1 ml fractions and lysed in Laemmli buffer as described above. % sucrose in each fraction was measured with a Bellingham+Stanley Abbe 60 Refractometer (Bellingham+Stanley, Tunbridge Wells, UK).

### Immunoprecipitation

THP-1 cells (10^7^) were lysed in 50 mM HEPES pH 7.5 (Sigma), 100 mM NaCl (Sigma), 10% glycerol, 0.5% NP-40 (Sigma), 0.5% CHAPS (Sigma), protease inhibitors (Roche, West Sussex, UK), phosphatase inhibitor cocktail 1, 2 (Sigma) and 300 μg/ml PMSF (Sigma) then passed through a 21 G needle. Insoluble material was removed by centrifugation and the supernatant pre-cleared with protein A-agarose (Sigma) for 2 h at 4°C. Pre-cleared supernatant was then incubated with anti-OPTN antibody (Sigma) for 30 min at 4°C. Protein A-agarose was added, incubated overnight at 4°C and then washed five times with ice-cold PBS. THP-1 supernatant was incubated with protein A-agarose overnight at 4°C and then washed five times with ice-cold PBS and used as a negative control.

### Immunoblot

Cells were lysed in Laemmli sample buffer containing β-mercaptoethanol (Sigma), protease inhibitors (Roche) and phosphatase inhibitors (Sigma). Samples were run on SDS-PAGE gels and transferred onto Hybond-P PVDF membranes (Amersham, Buckinghamshire, UK). Membranes were blocked in 5% non-fat milk then probed with OPTN (Sigma), actin (Sigma), EEA1 (Cell Signaling, Hitchin, UK), LAMP1 (Abcam, Cambridge, UK), GM130 (BD, Oxford, UK), Golgin-245 (Santa Cruz, Heidelberg, Germany) or GAPDH (Santa Cruz) for MDM/THP-1 cells or OPTN (Abcam) and TNF (Abcam) for BMDMs overnight at 4°C and anti-rabbit IgG-HRP (Cell Signaling) or anti-mouse IgG-HRP (GE Healthcare, Buckinghamshire, UK) for 1 h at room temperature. Bound antibody was detected using ECL Plus (Amersham), exposed to Hyperfilm ECL (Amersham), quantified and normalised to actin using ImageJ (NIH).

### Mass spectrometry

For liquid chromatography-tandem mass spectrometry (LC-MS/MS) analysis, proteins were separated by 10% SDS-PAGE under reducing conditions. Proteins were visualised by silver staining with ProteoSilver Plus (Sigma), bands were excised from both the OPTN-IP and control-IP gel lanes and processed for in-gel digestion and LC-MS/MS with the LTQ-Orbitrap mass spectrometer (Thermo Fisher Scientific, Loughborough, UK), as previously described (Mulvey et al., 2013). Raw MS files were analysed by the Mascot search engine 2.3.02 (Matrix Science, London, UK) and searched against a SwissProt human database 2013_10 (containing 39,696 entries including common contaminants). Mascot search analysis parameters included: trypsin enzyme specificity, allowance for 2 missed cleavages, peptide mass tolerance of 20 ppm for precursor ions and fragment mass tolerance of 0.8 Da. Oxidation (M) was selected as a variable modification and carbamidomethyl (C) was selected as a fixed modification.

### Lysosomal inhibition and TNF production

BMDMs were stimulated for 4 h with HkEc at a MOI of 20 plus either DMEM alone, or DMEM with 2.5 μM monensin (Sigma), 10 mM NH_4_Cl (Sigma), 100 μM chloroquine (Sigma), 2.5 μM brefeldin A (Merck) or 200 nM bafilomycin A (Sigma). Whole cell lysates were made and immunoblotted for TNF as described above.

### Intraperitoneal *E. coli* infection

*E. coli* NCTC 10418 was cultured in Luria-Bertani (LB) broth, washed and counted using a spectrophotometer. Nine- to twelve-week-old mice were injected intraperitoneally with serially diluted *E. coli* at 1×10^8^, 5×10^7^, 2.5×10^7^ and 1×10^7^ bacteria. Mice were weighed daily. Tail bleeds were collected for cytokine analysis at 48 h. Serum TNF levels were measured using a murine TNFα ELISA kit (Peprotech). Peritoneal washouts were harvested in cell dissociation buffer (Gibco) and analysed using flow cytometry.

### Zebrafish *Salmonella* infection

*Salmonella enterica* serovar Typhimurium was grown in LB broth and exposed to groups of 20 zebrafish larvae at 4 dpf at a final concentration of 5×10^8^ CFU/ml at 28.5°C. 1 nl (∼200 CFU) *Salmonella* was injected into the yolk sac of anesthetised 2-dpf embryos (Prajsnar et al., 2008). cDNA was synthesised with the High Capacity cDNA Reverse Transcription Kit (Applied Biosystems, Auckland, New Zealand). Morpholinos (GeneTools, LLC, Philomath, OR, USA) were designed to target the splice donor site after exon 1 of the *optn* gene (supplementary material Table S3). Morpholinos were injected into one- to four-cell-stage embryos at 1.0 pmol per embryo as previously described (Nasevicius and Ekker, 2000). Embryos were then injected with *Salmonella* at 2 dpf as above and incubated for observations at 28°C. qRT-PCR and primers used (supplementary material Table S3) were as previously described (Oehlers et al., 2011).

### *Citrobacter rodentium* colitis

*C. rodentium* strain ICC169 (gift from Gad Frankel, Imperial College London) was cultured in LB broth containing 50 µg/ml nalidixic acid. Nine- to twelve-week-old mice were gavaged with 200 µl of *Citrobacter* in PBS giving each mouse 2.5-4.5×10^9^ CFU of *Citrobacter*. Mice were weighed daily. After 2, 3 or 9 days, mice were culled, and blood from cardiac punctures, large bowel and spleens were collected. Cytokine levels were measured using the Mouse Proinflammatory Ultrasensitive plate (Meso Scale Discovery).

### Dextran sodium sulphate colitis

Nine- to twelve-week-old mice were given drinking water containing 2% DSS (MW 36,000-50,000) (MP Biomedicals, Cambridge, UK) for 7 days as previously described (Wirtz et al., 2007). The normal drinking water in the animal unit was used to make up the 2% DSS to minimise the effect of alteration in water taste on consumption of the DSS that results from autoclaving water. The DSS was changed with fresh DSS after 2 days and 5 days from the start of the experiment and was changed back to fresh drinking water in a new water bottle after 7 days. Mice were weighed daily, tested for faecal occult blood (Immunostics, Ocean, NJ, USA) and culled after 7, 14 and 21 days for collection of blood for cytokine analysis and large bowel for histology.

### Large bowel lamina propria cell isolation

Large bowels were cut longitudinally and washed in ice-cold PBS containing 100 U/ml penicillin, 100 µg/ml streptomycin (Gibco) to remove faeces. Epithelial cells were removed by incubation of each large bowel in 20 ml of predigestion solution [HBSS (Gibco) containing 10% FBS, 100 U/ml penicillin, 100 µg/ml streptomycin and 2 mM EDTA] at 37°C, 250 rpm for 1 h. Epithelial cells were passed through a 70 μm filter. The remaining lamina propria tissue was cut into 1 mm pieces and washed with PBS to remove EDTA. Lamina propria tissue was incubated in 20 ml digestion solution [HBSS containing 10% FBS, 100 U/ml penicillin, 100 µg/ml streptomycin, 30 mg collagenase (Sigma), 0.8 mg DNase I (Sigma) and 15 mg Dispase II (Sigma)] at 37°C, 250 rpm for 30 min, and vortexed for 20 s at the start, middle and end of incubation. Lamina propria cells were passed through a 70 μm filter, washed with PBS then stained for flow cytometry.

### Histology and immunohistochemistry

Large bowel tissue was fixed in 10% neutral buffered formalin (CellPath, Powys, UK) overnight then paraffin-embedded using a Leica TP1050 tissue processor. Sections were stained in VFM Harris’ hematoxylin (CellPath), differentiated in 0.2% acid alcohol and stained in Eosin Y (VWR) using a Leica ST4040 linear stainer and mounted in Pertex (Leica, Milton Keynes, UK). Colitis scoring of H&E-stained large bowel was performed blind with the following six parameters. Epithelial hyperplasia: 1, mild; 2, moderate; 3, severe. Crypt deformity/architectural distortion: 1, mild; 2, moderate, affecting >50%; 3, severe, near 100% surface. Ulceration: 1, small focal erosions; 2, small ulcers/multiple erosions; 3, large/deep transmural ulcers. Variation: 1, patchy inflammation; 2, >50% inflammation; 3, severe, near 100% inflammation. Inflammatory cell infiltrate: 1, few multifocal mononuclear cells; 2, several multifocal areas; 3, multiple transmural infiltrates. Goblet cell depletion: 1, mild/scattered depletion; 2, moderate/>50% depletion; 3, severe depletion. The anti-OPTN antibody (Sigma) was used to performed immunohistochemistry on available UCLH archival *OPTN*^low^ CD patient bowel biopsy samples and HC small bowel. After preliminary optimisation, optimal conditions were chosen based upon the criterion of background-free selective cellular labelling. Sections underwent automated dewaxing and endogenous peroxidase was blocked using 3-4% (v/v) hydrogen peroxide. The anti-OPTN antibody was used on the *OPTN*^low^ and HC small bowel samples at a dilution of 1:200 with 30 min incubation at ambient temperature following heat-induced epitope retrieval for 20 min using an EDTA-based (pH 9.0) epitope retrieval solution. Signal visualisation using the Bond Polymer Refine Detection Kit (DS9800) with DAB Enhancer (AR9432) was performed on the Bond-III automated staining platform (Leica). Cell nuclei were counterstained with haematoxylin. Slides were imaged with a Hamamatsu NanoZoomer 2.0-HT C9600 (Hamamatsu, Hertfordshire, UK).

### Confocal immunofluorescence microscopy

Cells were plated onto methanol-cleaned glass coverslips, stimulated then fixed in 4% formaldehyde, quenched, permeabilised and blocked in desalted human IgG. MDMs were stained with OPTN (gift from Folma Buss, University of Cambridge), GM130 (BD), EEA1 (BD), Alexa-Fluor^®^ 488-TNF/adalimumab (Abbvie, Berkshire, UK), Alexa-Fluor^®^ 546 anti-rabbit IgG (Invitrogen, Paisley, UK), Alexa-Fluor^®^ 488 anti-mouse IgG (Invitrogen) and DAPI (Invitrogen) in confocal buffer (PBS, 0.5% BSA, 0.1% saponin). BMDMs were stained with Alexa-Fluor^®^ 488-TNF (BD), EEA1 (Abcam) and GM130 (BD). Cells were imaged on a Leica TCS SPE confocal microscope. Colocalisation analysis was performed using ImageJ (NIH). The image calculator was used with the AND operator to generate an image of colocalised pixels for each *z*-stack, then the histogram function was used to quantify the total number of TNF, EEA1, GM130 and colocalised pixels.

### Flow cytometry

Cells were blocked in anti-CD16/CD32 (eBioscience) prior to staining with anti-CD11b-V450, CD19-PE or Gr1-PE, CD3-PE-Cy™7, CD45-PerCP-Cy™5.5 or CD19-PerCP-Cy™5.5, Ly-6C-APC (all from BD) and F4/80-FITC (eBioscience, Hatfield, UK). Lamina propria cells were incubated with the LIVE/DEAD^®^ stain (Invitrogen) prior to staining above. Cells were run on a BD LSRFortessa or LSR II after optimisation with compensation particles (BD) and analysed using FlowJo (Tree Star, Ashland, OR, USA).

### Autophagy assay

Whole cell lysates from BMDMs stimulated with bafilomycin A (Sigma) and HkEc at time points up to 24 h were immunoblotted for LC3B (Sigma) to investigate autophagy in BMDMs.

### Endoplasmic reticulum stress assay

BMDMs stimulated with HkEc for 4 h were immunoblotted for CHOP (Affinity BioReagents, Golden, CO, USA), GRP78/BiP (Santa Cruz) and GRP94 (Santa Cruz). Additionally, BMDMs were stimulated for 4 h with thapsigargin (Sigma), tunicamycin (Sigma) and bafilomycin A (Sigma) for mRNA. Total RNA was harvested and converted to cDNA as described above. PCR was performed on cDNA samples and digested with *Pst*I (Promega, Southampton, UK) restriction enzyme. Samples were run on a 2% high-performance MetaPhor™ Agarose (Lonza) gel, made as per the manufacturer's instructions and run at 4°C to separate the bands.

### Phagocytosis assay

10^5^ BMDMs were plated onto Corning 96-well special optics plates (Sigma) and incubated overnight to allow cells to adhere to the bottom of the plate. FITC-HkEc at an MOI of 20 was added to each well. 10 μl of 2.5 mg/ml Trypan blue was added to each well to quench the FITC at different time points. Fluorescence intensity was measured at an excitation wavelength of 485 nm and read at an emission wavelength of 520 nm using a FLUOstar Omega microplate reader (BMG LABTECH, Buckinghamshire, UK).

### Killing assay

2.5×10^5^ BMDMs/well were incubated in a 24-well plate overnight to allow cells to adhere. Adherent BMDMs were incubated overnight in media with no antibiotics to allow washing out of the antibiotics. BMDMs were incubated with *E. coli* in media containing no antibiotics at an MOI of 20 for 2 h to facilitate adequate phagocytosis of *E. coli*. BMDMs were incubated in media containing 300 μg/ml gentamicin for 1 h to kill extracellular *E. coli*. Cells were washed once in PBS to remove gentamicin and lysed with 1% Triton X-100 (BDH, Nottingham, UK). Remaining BMDMs were incubated in media containing 100 μg/ml gentamicin for further time points. Serial dilutions of lysed cells were plated on LB agar plates.

### Statistical analysis

Statistical significance was calculated using paired or unpaired two-tailed Student's *t*-test, one-way ANOVA with Bonferroni's multiple comparisons test, logrank or Fisher's exact test. Mean differences were considered significant when *P*<0.05.

## Supplementary Material

Supplementary Material

## References

[DMM020362C1] Albagha O. M. E., Visconti M. R., Alonso N., Langston A. L., Cundy T., Dargie R., Dunlop M. G., Fraser W. D., Hooper M. J., Isaia G. et al. (2010). Genome-wide association study identifies variants at CSF1, OPTN and TNFRSF11A as genetic risk factors for Paget's disease of bone. *Nat. Genet.* 42, 520-524. 10.1038/ng.562 20436471 PMC3217192

[DMM020362C2] Baumgart D. C. and Sandborn W. J. (2012). Crohn's disease. *Lancet* 380, 1590-1605. 10.1016/S0140-6736(12)60026-9 22914295

[DMM020362C3] Bedell V. M., Westcot S. E. and Ekker S. C. (2011). Lessons from morpholino-based screening in zebrafish. *Brief. Funct. Genomics* 10, 181-188. 10.1093/bfgp/elr021 21746693 PMC3144740

[DMM020362C4] Belzil V. V., Daoud H., Desjarlais A., Bouchard J.-P., Dupré N., Camu W., Dion P. A. and Rouleau G. A. (2011). Analysis of OPTN as a causative gene for amyotrophic lateral sclerosis. *Neurobiol. Aging* 32, 555.e13-555.e14. 10.1016/j.neurobiolaging.2010.10.001 21074290

[DMM020362C5] Bond L. M., Peden A. A., Kendrick-Jones J., Sellers J. R. and Buss F. (2011). Myosin VI and its binding partner optineurin are involved in secretory vesicle fusion at the plasma membrane. *Mol. Biol. Cell* 22, 54-65. 10.1091/mbc.E10-06-0553 21148290 PMC3016977

[DMM020362C6] Bosenko D. V., Zinkevich N. S., Tyler R. C., Link B. A. and Semina E. V. (2004). Sequence and expression of zebrafish optineurin gene suggests conserved function in vertebrate eye. *Invest. Ophthalmol. Vis. Sci.* 45, 4408.

[DMM020362C7] Chassaing B., Aitken J. D., Malleshappa M. and Vijay-Kumar M. (2014). Dextran sulfate sodium (DSS)-induced colitis in mice. *Curr. Protoc. Immunol.* 104, Unit 15 25 10.1002/0471142735.im1525s104 PMC3980572 24510619

[DMM020362C8] Chew T., Sewell G., O'Shea N., Bloom S., Segal A. and Smith A. (2014). LB-003 the role of optineurin in macrophage cytokine secretion and bowel inflammation. *Gut* 63 Suppl. 1, e2 10.1136/gutjnl-2014-307263.LB3

[DMM020362C9] Chi Z.-L., Akahori M., Obazawa M., Minami M., Noda T., Nakaya N., Tomarev S., Kawase K., Yamamoto T., Noda S. et al. (2010). Overexpression of optineurin E50K disrupts Rab8 interaction and leads to a progressive retinal degeneration in mice. *Hum. Mol. Genet.* 19, 2606-2615. 10.1093/hmg/ddq146 20388642 PMC2883340

[DMM020362C10] Claes A.-K., Steck N., Schultz D., Zähringer U., Lipinski S., Rosenstiel P., Geddes K., Philpott D. J., Heine H. and Grassl G. A. (2014). Salmonella enterica serovar Typhimurium DeltamsbB triggers exacerbated inflammation in Nod2 deficient mice. *PLoS ONE* 9, e113645 10.1371/journal.pone.0113645 25423082 PMC4244092

[DMM020362C11] Collins J. W., Keeney K. M., Crepin V. F., Rathinam V. A., Fitzgerald K. A., Finlay B. B. and Frankel G. (2014). Citrobacter rodentium: infection, inflammation and the microbiota. *Nat. Rev. Microbiol.* 12, 612-623. 10.1038/nrmicro3315 25088150

[DMM020362C12] de Bruyn M., Machiels K., Vandooren J., Lemmens B., Van Lommel L., Breynaert C., Van der Goten J., Staelens D., Billiet T., De Hertogh G. et al. (2014). Infliximab restores the dysfunctional matrix remodeling protein and growth factor gene expression in patients with inflammatory bowel disease. *Inflamm. Bowel Dis.* 20, 339-352. 10.1097/01.MIB.0000438430.15553.90 24378596

[DMM020362C13] Del Bo R., Tiloca C., Pensato V., Corrado L., Ratti A., Ticozzi N., Corti S., Castellotti B., Mazzini L., Soraru G. et al. (2011). Novel optineurin mutations in patients with familial and sporadic amyotrophic lateral sclerosis. *J. Neurol. Neurosurg. Psychiatry* 82, 1239-1243. 10.1136/jnnp.2011.242313 21613650

[DMM020362C14] del Toro D., Alberch J., Lazaro-Dieguez F., Martin-Ibanez R., Xifro X., Egea G. and Canals J. M. (2009). Mutant huntingtin impairs post-Golgi trafficking to lysosomes by delocalizing optineurin/Rab8 complex from the Golgi apparatus. *Mol. Biol. Cell* 20, 1478-1492. 10.1091/mbc.E08-07-0726 19144827 PMC2649260

[DMM020362C15] Dhillon S. S., Fattouh R., Elkadri A., Xu W., Murchie R., Walters T., Guo C., Mack D., Huynh H. Q., Baksh S. et al. (2014). Variants in NADPH oxidase complex components determine susceptibility to very early onset inflammatory bowel disease. *Gastroenterology* 147, 680-689.e2. 10.1053/j.gastro.2014.06.005 24931457

[DMM020362C16] Dippold H. C., Ng M. M., Farber-Katz S. E., Lee S.-K., Kerr M. L., Peterman M. C., Sim R., Wiharto P. A., Galbraith K. A., Madhavarapu S. et al. (2009). GOLPH3 bridges phosphatidylinositol-4- phosphate and actomyosin to stretch and shape the Golgi to promote budding. *Cell* 139, 337-351. 10.1016/j.cell.2009.07.052 19837035 PMC2779841

[DMM020362C17] Dunn D. L., Barke R. A., Knight N. B., Humphrey E. W. and Simmons R. L. (1985). Role of resident macrophages, peripheral neutrophils, and translymphatic absorption in bacterial clearance from the peritoneal cavity. *Infect. Immun.* 49, 257-264. 3894229 10.1128/iai.49.2.257-264.1985 PMC262007

[DMM020362C18] Farthing M. J. G. (2004). Bugs and the gut: an unstable marriage. *Best Pract. Res. Clin. Gastroenterol.* 18, 233-239. 10.1016/j.bpg.2003.11.001 15123066

[DMM020362C19] Franke A., McGovern D. P. B., Barrett J. C., Wang K., Radford-Smith G. L., Ahmad T., Lees C. W., Balschun T., Lee J., Roberts R. et al. (2010). Genome-wide meta-analysis increases to 71 the number of confirmed Crohn's disease susceptibility loci. *Nat. Genet.* 42, 1118-1125. 10.1038/ng.717 21102463 PMC3299551

[DMM020362C20] Ginhoux F. and Jung S. (2014). Monocytes and macrophages: developmental pathways and tissue homeostasis. *Nat. Rev. Immunol.* 14, 392-404. 10.1038/nri3671 24854589

[DMM020362C21] Gleason C. E., Ordureau A., Gourlay R., Arthur J. S. C. and Cohen P. (2011). Polyubiquitin binding to optineurin is required for optimal activation of TANK-binding kinase 1 and production of interferon beta. *J. Biol. Chem.* 286, 35663-35674. 10.1074/jbc.M111.267567 21862579 PMC3195586

[DMM020362C22] Goncalves N. S., Ghaem-Maghami M., Monteleone G., Frankel G., Dougan G., Lewis D. J. M., Simmons C. P. and MacDonald T. T. (2001). Critical role for tumor necrosis factor alpha in controlling the number of lumenal pathogenic bacteria and immunopathology in infectious colitis. *Infect. Immun.* 69, 6651-6659. 10.1128/IAI.69.11.6651-6659.2001 11598034 PMC100039

[DMM020362C23] Hall L. J., Faivre E., Quinlan A., Shanahan F., Nally K. and Melgar S. (2011). Induction and activation of adaptive immune populations during acute and chronic phases of a murine model of experimental colitis. *Dig. Dis. Sci.* 56, 79-89. 10.1007/s10620-010-1240-3 20467900

[DMM020362C24] Hugot J.-P., Chamaillard M., Zouali H., Lesage S., Cézard J.-P., Belaiche J., Almer S., Tysk C., O'Morain C. A., Gassull M. et al. (2001). Association of NOD2 leucine-rich repeat variants with susceptibility to Crohn's disease. *Nature* 411, 599-603. 10.1038/35079107 11385576

[DMM020362C25] Ito H., Fujita K., Nakamura M., Wate R., Kaneko S., Sasaki S., Yamane K., Suzuki N., Aoki M., Shibata N. et al. (2011). Optineurin is co-localized with FUS in basophilic inclusions of ALS with FUS mutation and in basophilic inclusion body disease. *Acta Neuropathol.* 121, 555-557. 10.1007/s00401-011-0809-z 21327942

[DMM020362C26] Jostins L. Ripke S. Weersma R. K. Duerr R. H. McGovern D. P. Hui K. Y. Lee J. C. Schumm L. P. Sharma Y. Anderson C. A. et al. (2012). Host-microbe interactions have shaped the genetic architecture of inflammatory bowel disease. *Nature* 491, 119-124. 10.1038/nature11582 23128233 PMC3491803

[DMM020362C27] Khor B., Gardet A. and Xavier R. J. (2011). Genetics and pathogenesis of inflammatory bowel disease. *Nature* 474, 307-317. 10.1038/nature10209 21677747 PMC3204665

[DMM020362C28] Kim Y.-G., Kamada N., Shaw M. H., Warner N., Chen G. Y., Franchi L. and Núñez G. (2011). The Nod2 sensor promotes intestinal pathogen eradication via the chemokine CCL2-dependent recruitment of inflammatory monocytes. *Immunity* 34, 769-780. 10.1016/j.immuni.2011.04.013 21565531 PMC3103637

[DMM020362C29] Kobayashi K. S., Chamaillard M., Ogura Y., Henegariu O., Inohara N., Nuñez G. and Flavell R. A. (2005). Nod2-dependent regulation of innate and adaptive immunity in the intestinal tract. *Science* 307, 731-734. 10.1126/science.1104911 15692051

[DMM020362C30] Livak K. J. and Schmittgen T. D. (2001). Analysis of relative gene expression data using real-time quantitative PCR and the 2(-Delta Delta C(T)) Method. *Methods* 25, 402-408. 10.1006/meth.2001.1262 11846609

[DMM020362C31] Manderson A. P., Kay J. G., Hammond L. A., Brown D. L. and Stow J. L. (2007). Subcompartments of the macrophage recycling endosome direct the differential secretion of IL-6 and TNFalpha. *J. Cell Biol.* 178, 57-69. 10.1083/jcb.200612131 17606866 PMC2064421

[DMM020362C32] Mankouri J., Fragkoudis R., Richards K. H., Wetherill L. F., Harris M., Kohl A., Elliott R. M. and Macdonald A. (2010). Optineurin negatively regulates the induction of IFNbeta in response to RNA virus infection. *PLoS Pathog.* 6, e1000778 10.1371/journal.ppat.1000778 20174559 PMC2824764

[DMM020362C33] Marino M. W., Dunn A., Grail D., Inglese M., Noguchi Y., Richards E., Jungbluth A., Wada H., Moore M., Williamson B. et al. (1997). Characterization of tumor necrosis factor-deficient mice. *Proc. Natl. Acad. Sci. USA* 94, 8093-8098. 10.1073/pnas.94.15.8093 9223320 PMC21562

[DMM020362C34] Marks D. J. B., Harbord M. W. N., MacAllister R., Rahman F. Z., Young J., Al-Lazikani B., Lees W., Novelli M., Bloom S. and Segal A. W. (2006). Defective acute inflammation in Crohn's disease: a clinical investigation. *Lancet* 367, 668-678. 10.1016/S0140-6736(06)68265-2 16503465

[DMM020362C35] Marks D. J. B., Miyagi K., Rahman F. Z., Novelli M., Bloom S. L. and Segal A. W. (2009). Inflammatory bowel disease in CGD reproduces the clinicopathological features of Crohn's disease. *Am. J. Gastroenterol.* 104, 117-124. 10.1038/ajg.2008.72 19098859

[DMM020362C36] Maruyama H., Morino H., Ito H., Izumi Y., Kato H., Watanabe Y., Kinoshita Y., Kamada M., Nodera H., Suzuki H. et al. (2010). Mutations of optineurin in amyotrophic lateral sclerosis. *Nature* 465, 223-226. 10.1038/nature08971 20428114

[DMM020362C37] Medzhitov R. (2008). Origin and physiological roles of inflammation. *Nature* 454, 428-435. 10.1038/nature07201 18650913

[DMM020362C38] Millecamps S., Boillée S., Chabrol E., Camu W., Cazeneuve C., Salachas F., Pradat P.-F., Danel-Brunaud V., Vandenberghe N., Corcia P. et al. (2011). Screening of OPTN in French familial amyotrophic lateral sclerosis. *Neurobiol. Aging* 32, 557.e11-557.e13. 10.1016/j.neurobiolaging.2010.11.005 21220178

[DMM020362C39] Mulvey C. M., Tudzarova S., Crawford M., Williams G. H., Stoeber K. and Godovac-Zimmermann J. (2013). Subcellular proteomics reveals a role for nucleo-cytoplasmic trafficking at the DNA replication origin activation checkpoint. *J. Proteome Res.* 12, 1436-1453. 10.1021/pr3010919 23320540 PMC4261602

[DMM020362C40] Munitic I., Giardino Torchia M. L., Meena N. P., Zhu G., Li C. C. and Ashwell J. D. (2013). Optineurin insufficiency impairs IRF3 but not NF-kappaB activation in immune cells. *J. Immunol.* 191, 6231-6240. 10.4049/jimmunol.1301696 24244017 PMC3886234

[DMM020362C41] Murray R. Z., Kay J. G., Sangermani D. G. and Stow J. L. (2005). A role for the phagosome in cytokine secretion. *Science* 310, 1492-1495. 10.1126/science.1120225 16282525

[DMM020362C42] Murthy A., Li Y., Peng I., Reichelt M., Katakam A. K., Noubade R., Roose-Girma M., DeVoss J., Diehl L., Graham R. R. et al. (2014). A Crohn's disease variant in Atg16l1 enhances its degradation by caspase 3. *Nature* 506, 456-462. 10.1038/nature13044 24553140

[DMM020362C43] Naito Y., Takagi T., Handa O., Ishikawa T., Nakagawa S., Yamaguchi T., Yoshida N., Minami M., Kita M., Imanishi J. et al. (2003). Enhanced intestinal inflammation induced by dextran sulfate sodium in tumor necrosis factor-alpha deficient mice. *J. Gastroenterol. Hepatol.* 18, 560-569. 10.1046/j.1440-1746.2003.03034.x 12702049

[DMM020362C65] Nasevicius A. and Ekker S. C. (2000). Effective targeted gene ‘knockdown’ in zebrafish. *Nat. Genet.* 26, 216-220. 10.1038/79951 11017081

[DMM020362C44] Oehlers S. H., Flores M. V., Chen T., Hall C. J., Crosier K. E. and Crosier P. S. (2011). Topographical distribution of antimicrobial genes in the zebrafish intestine. *Dev. Comp. Immunol.* 35, 385-391. 10.1016/j.dci.2010.11.008 21093479

[DMM020362C45] Ogura Y., Bonen D. K., Inohara N., Nicolae D. L., Chen F. F., Ramos R., Britton H., Moran T., Karaliuskas R., Duerr R. H. et al. (2001). A frameshift mutation in NOD2 associated with susceptibility to Crohn's disease. *Nature* 411, 603-606. 10.1038/35079114 11385577

[DMM020362C46] Perse M. and Cerar A. (2012). Dextran sodium sulphate colitis mouse model: traps and tricks. *J. Biomed. Biotechnol.* 2012, 718617 10.1155/2012/718617 22665990 PMC3361365

[DMM020362C47] Prajsnar T. K., Cunliffe V. T., Foster S. J. and Renshaw S. A. (2008). A novel vertebrate model of Staphylococcus aureus infection reveals phagocyte-dependent resistance of zebrafish to non-host specialized pathogens. *Cell Microbiol.* 10, 2312-2325. 10.1111/j.1462-5822.2008.01213.x 18715285

[DMM020362C48] Rahman F. Z., Smith A. M., Hayee B., Marks D. J. B., Bloom S. L. and Segal A. W. (2010). Delayed resolution of acute inflammation in ulcerative colitis is associated with elevated cytokine release downstream of TLR4. *PLoS ONE* 5, e9891 10.1371/journal.pone.0009891 20360984 PMC2847519

[DMM020362C49] Rezaie T., Child A., Hitchings R., Brice G., Miller L., Coca-Prados M., Héon E., Krupin T., Ritch R., Kreutzer D. et al. (2002). Adult-onset primary open-angle glaucoma caused by mutations in optineurin. *Science* 295, 1077-1079. 10.1126/science.1066901 11834836

[DMM020362C50] Sahlender D. A., Roberts R. C., Arden S. D., Spudich G., Taylor M. J., Luzio J. P., Kendrick-Jones J. and Buss F. (2005). Optineurin links myosin VI to the Golgi complex and is involved in Golgi organization and exocytosis. *J. Cell Biol.* 169, 285-295. 10.1083/jcb.200501162 15837803 PMC2171882

[DMM020362C51] Schreiber H. A., Loschko J., Karssemeijer R. A., Escolano A., Meredith M. M., Mucida D., Guermonprez P. and Nussenzweig M. C. (2013). Intestinal monocytes and macrophages are required for T cell polarization in response to Citrobacter rodentium. *J. Exp. Med.* 210, 2025-2039. 10.1084/jem.20130903 24043764 PMC3782042

[DMM020362C52] Segal A. W. and Loewi G. (1976). Neutrophil dysfunction in Crohn's disease. *Lancet* 308, 219-221. 10.1016/S0140-6736(76)91024-2 59239

[DMM020362C53] Sewell G. W., Marks D. J. B. and Segal A. W. (2009). The immunopathogenesis of Crohn's disease: a three-stage model. *Curr. Opin. Immunol.* 21, 506-513. 10.1016/j.coi.2009.06.003 19665880 PMC4529487

[DMM020362C54] Sewell G. W., Rahman F. Z., Levine A. P., Jostins L., Smith P. J., Walker A. P., Bloom S. L., Segal A. W. and Smith A. M. (2012). Defective tumor necrosis factor release from Crohn's disease macrophages in response to toll-like receptor activation: relationship to phenotype and genome-wide association susceptibility loci. *Inflamm. Bowel Dis.* 18, 2120-2127. 10.1002/ibd.22952 22434667 PMC3532612

[DMM020362C55] Shurety W., Merino-Trigo A., Brown D., Hume D. A. and Stow J. L. (2000). Localization and post-Golgi trafficking of tumor necrosis factor-alpha in macrophages. *J. Interferon Cytokine Res.* 20, 427-438. 10.1089/107999000312379 10805378

[DMM020362C56] Skarnes W. C., Rosen B., West A. P., Koutsourakis M., Bushell W., Iyer V., Mujica A. O., Thomas M., Harrow J., Cox T. et al. (2011). A conditional knockout resource for the genome-wide study of mouse gene function. *Nature* 474, 337-342. 10.1038/nature10163 21677750 PMC3572410

[DMM020362C57] Smith A. M., Rahman F. Z., Hayee B., Graham S. J., Marks D. J. B., Sewell G. W., Palmer C. D., Wilde J., Foxwell B. M. J., Gloger I. S. et al. (2009). Disordered macrophage cytokine secretion underlies impaired acute inflammation and bacterial clearance in Crohn's disease. *J. Exp. Med.* 206, 1883-1897. 10.1084/jem.20091233 19652016 PMC2737162

[DMM020362C58] Smith A. M., Sewell G. W., Levine A. P., Chew T. S., Dunne J., O'Shea N. R., Smith P. J., Harrison P. J., Macdonald C. M., Bloom S. L. et al. (2015). Disruption of macrophage pro-inflammatory cytokine release in Crohn's disease is associated with reduced optineurin expression in a subset of patients. *Immunology* 144, 45-55. 10.1111/imm.12338 24943399 PMC4264909

[DMM020362C59] Solski J. A., Williams K. L., Yang S., Nicholson G. A. and Blair I. P. (2012). Mutation analysis of the optineurin gene in familial amyotrophic lateral sclerosis. *Neurobiol. Aging* 33, 210.e9-210.e10. 10.1016/j.neurobiolaging.2011.09.023 22015311

[DMM020362C60] Tumer Z., Bertelsen B., Gredal O., Magyari M., Nielsen K. C., Lucamp, Grønskov K. and Brøndum-Nielsen K. (2012). Novel heterozygous nonsense mutation of the OPTN gene segregating in a Danish family with ALS. *Neurobiol. Aging* 33, 208.e1-208.e5. 10.1016/j.neurobiolaging.2011.07.001 21852022

[DMM020362C61] Wild P., Farhan H., McEwan D. G., Wagner S., Rogov V. V., Brady N. R., Richter B., Korac J., Waidmann O., Choudhary C. et al. (2011). Phosphorylation of the autophagy receptor optineurin restricts salmonella growth. *Science* 333, 228-233. 10.1126/science.1205405 21617041 PMC3714538

[DMM020362C62] Wirtz S., Neufert C., Weigmann B. and Neurath M. F. (2007). Chemically induced mouse models of intestinal inflammation. *Nat. Protoc.* 2, 541-546. 10.1038/nprot.2007.41 17406617

[DMM020362C63] Xu Y., Hunt N. H. and Bao S. (2007). The correlation between proinflammatory cytokines, MAdCAM-1 and cellular infiltration in the inflamed colon from TNF-alpha gene knockout mice. *Immunol. Cell Biol.* 85, 633-639. 10.1038/sj.icb.7100112 17768420

[DMM020362C64] Yan D., Wang X., Luo L., Cao X. and Ge B. (2012). Inhibition of TLR signaling by a bacterial protein containing immunoreceptor tyrosine-based inhibitory motifs. *Nat. Immunol.* 13, 1063-1071. 10.1038/ni.2417 23001144

