# Supplementary material for: Optineurin deficiency in mice contributes to impaired cytokine secretion and neutrophil recruitment in bacteria-driven colitis
Source: Dis Model Mech. 2015 Aug 1;8(8):817–29. doi: 10.1242/dmm.020362 (PMC4527293; doi:10.1242/dmm.020362)
Supplement: Supplementary Material [file supp_8_8_817__index.html]

Supplementary Material 

# Optineurin deficiency contributes to impaired cytokine secretion and neutrophil recruitment in bacteria driven colitis

## DMM020362 Supplementary Material

- Supplementary Material
