# Supplementary material for: Optineurin deficiency in mice contributes to impaired cytokine secretion and neutrophil recruitment in bacteria-driven colitis
Source: Dis Model Mech. 2015 Aug 1;8(8):817–29. doi: 10.1242/dmm.020362 (PMC4527293; doi:10.1242/dmm.020362)
Supplement: Supplementary Material [file supp_020362_DMM020362supp.pdf]

SUPPLEMENTARY TABLES

Table S1 Proteins identified in the OPTN immunoprecipitation on mass spectrometry localise OPTN to the Golgi*.

Protein	Symbol	Mascot Protein Score	Mascot Expect Value	Peptides Identified	Protein Coverage %
Complement C4-B	C4B	1240	3.70E-120	22	18.4
Isoform 2 of Unconventional myosin-XVIIIa	MYO18A	1182	2.50E-114	20	18.6
Isoform 2 of Unconventional myosin-Ic	MYO1C	924	1.50E-88	17	27
Isoform 2 of Fibrinogen alpha chain	FGA	854	1.70E-81	15	32.1
Isoform 2 of Optineurin	OPTN	822	2.60E-78	14	32.7
High affinity immunoglobulin gamma Fc receptor I	FCGR1A	625	1.30E-58	11	28.9
Actin, cytoplasmic 2	ACTG1	633	1.80E-59	10	41.9
Isoform 2 of Myosin-10	MYH10	601	3.10E-56	10	5.7
Structural maintenance of chromosomes protein 4	SMC4	597	8.00E-56	10	11.3
Apolipoprotein B-100	APOB	428	5.70E-39	10	2.9
Isoform 6 of Unconventional myosin-VI	MYO6	546	9.70E-51	9	10.1
Isoform 2 of Myosin-14	MYH14	538	6.30E-50	9	4.5
Isoform Beta-1 of DNA topoisomerase 2-beta	TOP2B	401	3.50E-36	8	6.3
Complement factor H	CFH	389	4.80E-35	8	8.9
Valine--tRNA ligase	VARS	453	2.00E-41	6	8.5
Protein S100-A8	S100A8	311	2.90E-27	6	49.5
Isoform 2 of Programmed cell death 6-interacting protein	PDCD6IP	302	2.30E-26	6	8.4
Isoform 2 of Clusterin	CLU	300	4.20E-26	6	15.8
Protein-glutamine gamma-glutamyltransferase K	TGM1	277	8.50E-24	6	10.2
Isoform 1 of Protein POF1B	POF1B	260	4.50E-22	6	11.4
Unconventional myosin-If	MYO1F	333	2.10E-29	5	6.3
Protein S100-A9	S100A9	317	8.30E-28	5	51.8
Isoform 2 of Caprin-1	CAPRIN1	298	6.20E-26	5	8.4
Pentatricopeptide repeat domain-containing protein 3, mitochondrial	PTCD3	297	8.80E-26	5	9.1
Engulfment and cell motility protein 1	ELMO1	294	1.60E-25	5	11.1
Caspase-14	CASP14	285	1.40E-24	5	21.9
Coatomer subunit delta	ARCN1	257	8.50E-22	5	14.1
Isoform 2 of Protein flightless-1 homolog	FLII	255	1.20E-21	5	7.4
Structural maintenance of chromosomes protein 3	SMC3	249	5.40E-21	5	7.1
DNA replication licensing factor MCM4	MCM4	245	1.10E-20	5	8

*Only proteins that have been identified with five or more peptides, which are not found in the control sample are included.

Table S2 Gene Ontology analysis of proteins identified in the OPTN immunoprecipitation shows association with intracellular vesicles and the cytoskeleton.

GO Term	Go Term Name	Count	%	p value	Benjamini	Genes
GO:0016459	myosin complex	6	20.0	0.00000018	0.00003065	MYO6, MYO1C, MYO1F, MYH14, MYO18A, MYH10
GO:0015629	actin cytoskeleton	7	23.3	0.00001328	0.00115430	ACTG1, MYO6, MYO1C, MYO1F, MYH14, MYO18A, MYH10
GO:0043232	intracellular non-membrane-bounded organelle	16	53.3	0.00003096	0.00179392	MYO6, MYO1C, S100A9, MYO1F, MCM4, SMC3, SMC4, ELMO1 ACTG1, TGM1, FLII, MYH14, PDCD6IP, TOP2B, MYO18A, MYH10
GO:0043228	non-membrane-bounded organelle	16	53.3	0.00003096	0.00179392	MYO6, MYO1C, S100A9, MYO1F, MCM4, SMC3, SMC4, ELMO1, ACTG1, TGM1, FLII, MYH14, PDCD6IP, TOP2B, MYO18A, MYH10
GO:0005856	cytoskeleton	12	40.0	0.00003727	0.00162012	ACTG1, MYO6, MYO1C, TGM1, FLII, MYO1F, MYH14, PDCD6IP, MYO18A, SMC3, MYH10, ELMO1
GO:0016461	unconventional myosin complex	3	10.0	0.00008303	0.00288549	MYO6, MYO1C, MYO1F
GO:0031941	filamentous actin	3	10.0	0.00041103	0.01185145	MYO6, MYO1C, MYO1F
GO:0044430	cytoskeletal part	9	30.0	0.00042766	0.01057625	MYO6, MYO1C, FLII, MYO1F, MYH14, PDCD6IP, MYO18A, SMC3, MYH10
GO:0044433	cytoplasmic vesicle part	5	16.7	0.00051546	0.01115140	APOB, MYO6, FGA, ARCN1, CLU
GO:0016023	cytoplasmic membrane-bounded vesicle	7	23.3	0.00068045	0.01307366	APOB, MYO6, FGA, ARCN1, CLU, PDCD6IP, OPTN
GO:0031988	membrane-bounded vesicle	7	23.3	0.00080607	0.01393329	APOB, MYO6, FGA, ARCN1, CLU, PDCD6IP, OPTN
GO:0031410	cytoplasmic vesicle	7	23.3	0.00152299	0.02382103	APOB, MYO6, FGA, ARCN1, CLU, PDCD6IP, OPTN
GO:0031982	vesicle	7	23.3	0.00189513	0.02713060	APOB, MYO6, FGA, ARCN1, CLU, PDCD6IP, OPTN
GO:0043233	organelle lumen	11	36.7	0.00200579	0.02651576	APOB, MYO6, MYO1C, FGA, CLU, S100A9, FLII, TOP2B, MYO18A, MCM4, SMC3
GO:0031974	membrane-enclosed lumen	11	36.7	0.00233124	0.02859114	APOB, MYO6, MYO1C, FGA, CLU, S100A9, FLII, TOP2B, MYO18A, MCM4, SMC3
GO:0005884	actin filament	3	10.0	0.00310731	0.03545710	MYO6, MYO1C, MYO1F
GO:0005938	cell cortex	4	13.3	0.00313077	0.03352562	ACTG1, MYO6, MYO1F, MYH10
GO:0031983	vesicle lumen	3	10.0	0.00389765	0.03918319	APOB, FGA, CLU

Table S3 Primers, probes and morpholinos used.

Gene	Primer pair	Primer sequence	Size (bp)
		Mouse genotyping primers	
Wildtype *Optn*	Optn_47570 F	5'-ACCACACGATGGCTCACAAC-3'	435
	Optn_47570 R	5'-GTGTCTCAAAACAACAAATTCTCCC-3'	
Mutant *Optn*	Optn_47570 F	5'-ACCACACGATGGCTCACAAC-3'	238
	CAS_R1_Term x	5'-TCGTGGTATCGTTATGCGCC-3'	
Wildtype *Nod2*	oIMR4112 F	5'-ACAGAGATGCCGACACCATACTG-3'	370
	oIMR4113 R	5'-TGGAGAAGGTTGAAGAGCAGAGTC-3'	
Mutant *Nod2*	oIMR4114 F	5'-TGACTGTGGCTAATGTCCTTTGTG-3'	1000
	oIMR6955 R	5'-TTCTATCGCCTTCTTGACGAGTTC-3'	
		Mouse qRT-PCR primers	
Optn	mOptn2 F	5'-GAGCAGCTGGCCTTGCA-3'	73
	mOptn2 R	5'-ACTGTCTACTGCCTCCCTCTTCA-3'	
Tnf	mTnf F	5'-CTGAACTTCGGGGTGATCGG-3'	122
	mTnf R	5'-GGCTTGTCACTCGAATTTTGAGA-3'	
Cxcl1	mCxcl1 F	5'-ACTGCACCCAAACCGAAGTC-3'	114
	mCxcl1 R	5'-TGGGGACACCTTTTAGCATCTT-3'	
Il6	mIL6 F	5'-CGCTATGAAGTTCCTCTCTGCAA-3'	64
	mIL6 R	5'-CACCAGCATCAGTCCCAAGAA-3'	
Il10	mIL10 F	5'-GCTGGACAACATACTGCTAACC-3'	78
	mIL10 R	5'-ATTTCCGATAAGGCTTGGCAA-3'	
Ppia	mPpia F	5'-GGGCCGCGTCTCCTTT-3'	86
	mPpia R	5'-ATCCTTTCTCTCCAGTGCTCAGA-3'	
Mcm10	mMcm10 F	5'-TCCCAGCCAAGAGAAAACCA-3'	115
	mMcm10 R	5'-CCCTGGAGGCTGTTTAATTG-3'	
Ccdc3	mCcdc3 F	5'-CTACTTCTCCTGCCACTCCC-3'	93
	mCcdc3 R	5'-CCGTGTGGCAAGAGATTGTA-3'	
		Mouse RT-PCR primers	
Optn	mOptn ex2-4 F	5'-CTGACTGAGAAGGGGGACAG-3'	367
	mOptn ex2-4 R	5'-ACCTGTAGCCACCTGTGAGG-3'	

Gene	Primer pair	Primer sequence	Size (bp)
		Zebrafish morpholinos (MO)	
optn	*optn* MO	5'-AGAGCCTCTGTGGGATGCATATAAT-3'	-
control	control MO	5'-CCTCTTACCTCAGTTACAATTTATA-3'	
		Zebrafish qRT-PCR primers	
optn	optn qFw1163	5'-TCGATGAGATGAAGATGGAACTATTC-3'	77
	optn qRv1239	5'-TTCTGCCTGTGCCTGGAAA-3'	
ef1alpha	ef1alpha qFw	5'-TGCCTTCGTCCCAATTTCAG-3'	101
	ef1alpha qRv	5'-TACCCTCCTTGCGCTCAATC-3'	

SUPPLEMENTARY FIGURES

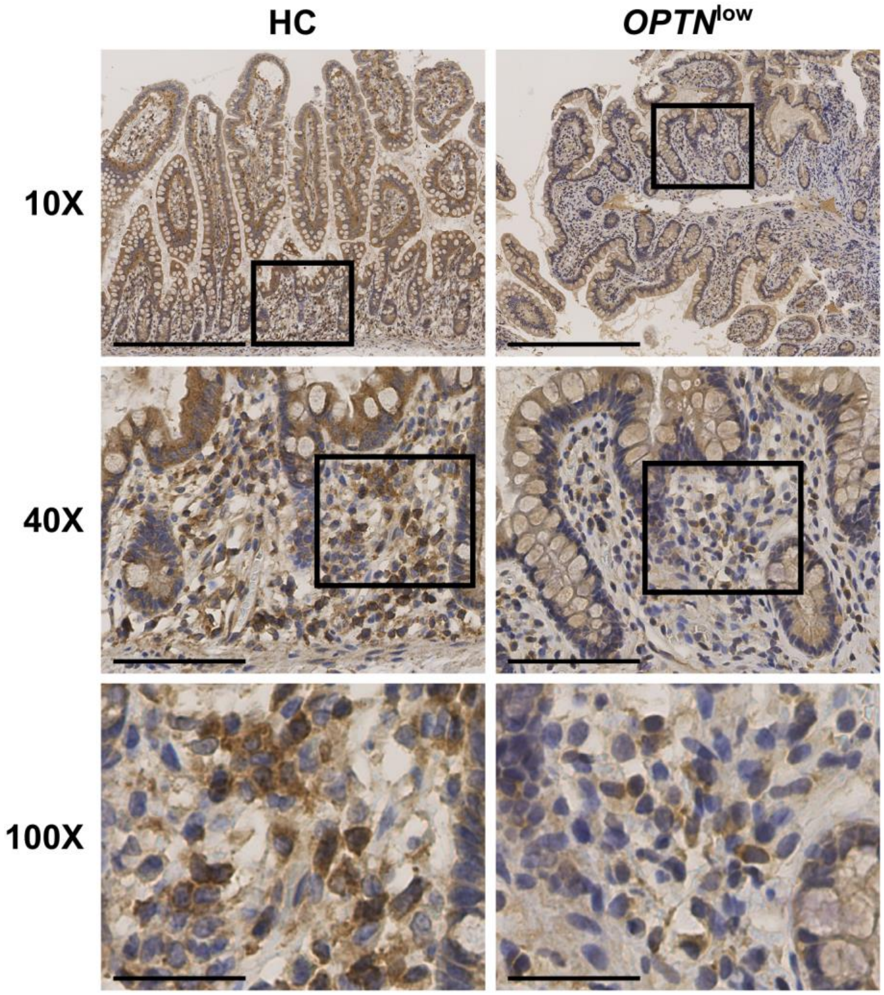

Figure S1 OPTN immunohistochemistry in small bowel samples in an *OPTN*^{low} CD patient and HC. Representative immunohistochemistry staining for OPTN in human small bowel shows possible weaker staining of OPTN in cells of the lamina propria that histologically resemble macrophages and B cells, in an *OPTN*^{low} CD patient compared to a HC. (Upper panels 10× magnification, scale bar 400 µm; middle 40×, 100 µm; lower 100×, 40 µm).

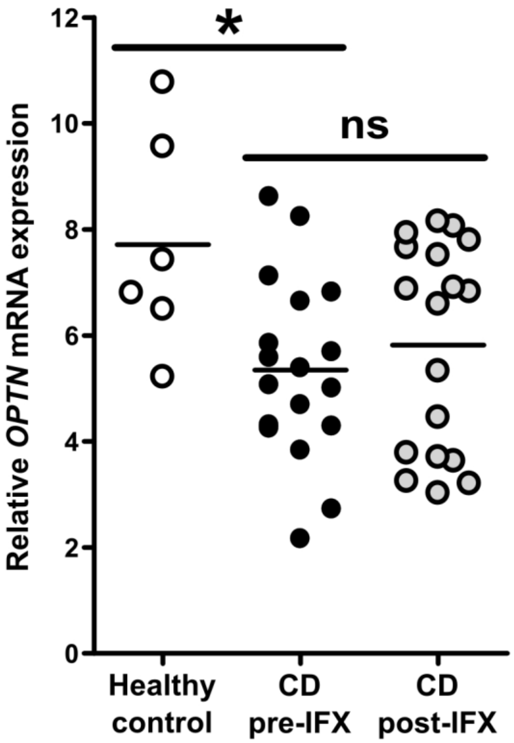

Figure S2 *OPTN* expression is reduced in the ileum of Crohn's disease patients. *OPTN* expression in the ileum was significantly lower in CD patients compared to healthy controls. *OPTN* expression in the ileum was minimally altered with infliximab anti-TNF treatment. Data from GSE 16879, de Bruyn et al. 2014. Results shown as dot plot plus mean (black line) (ns, non-significant, *p<0.05; two-tailed, unpaired and paired *t* test).

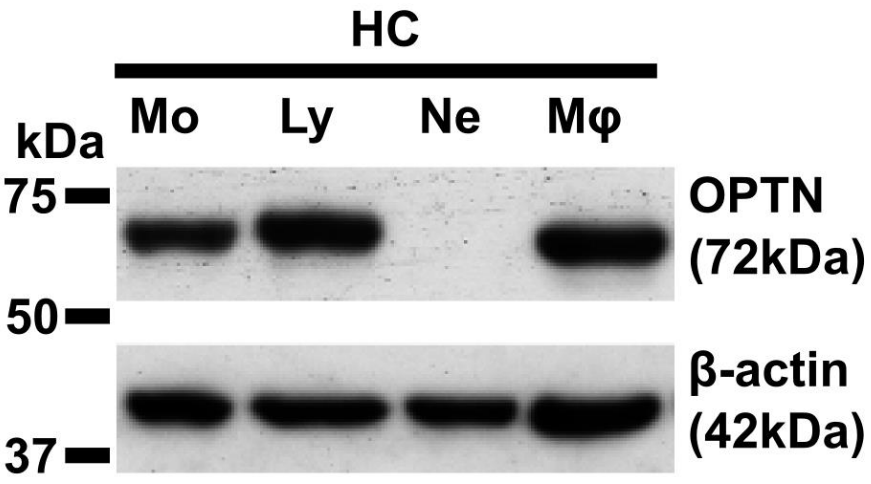

Figure S3 OPTN is expressed in human monocytes, lymphocytes and macrophages but is not detectable in neutrophils. Immunoblot for OPTN in human monocytes (Mo), lymphocytes (Ly), neutrophils (Ne) and macrophages (Mφ) with actin loading control in a HC.

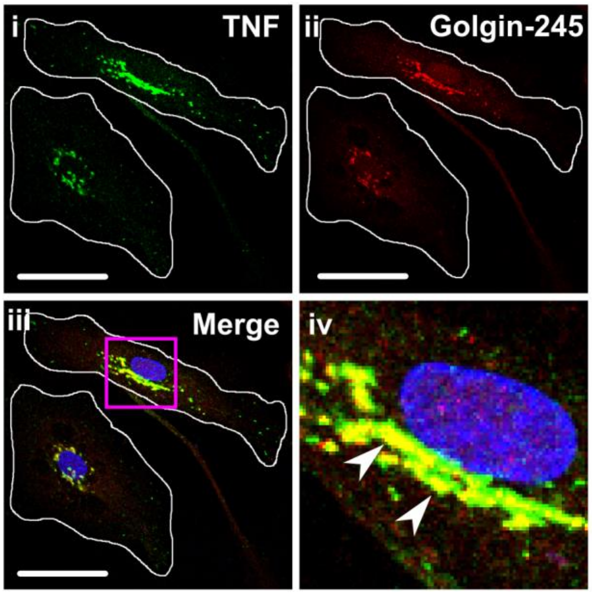

Figure S4 TNF localises to the Golgi complex in MDM. Confocal microscopy of MDM (white outline) after stimulation with HkEc for 4 hours were stained for (i) TNF, (ii) Golgin-245 (red) and (iii) nuclei. (iii and iv) merged image demonstrates colocalisation of TNF and Golgin-245 (white arrows) in the Golgi complex (image i, ii, iii at 63×, scale bar 20 µm).

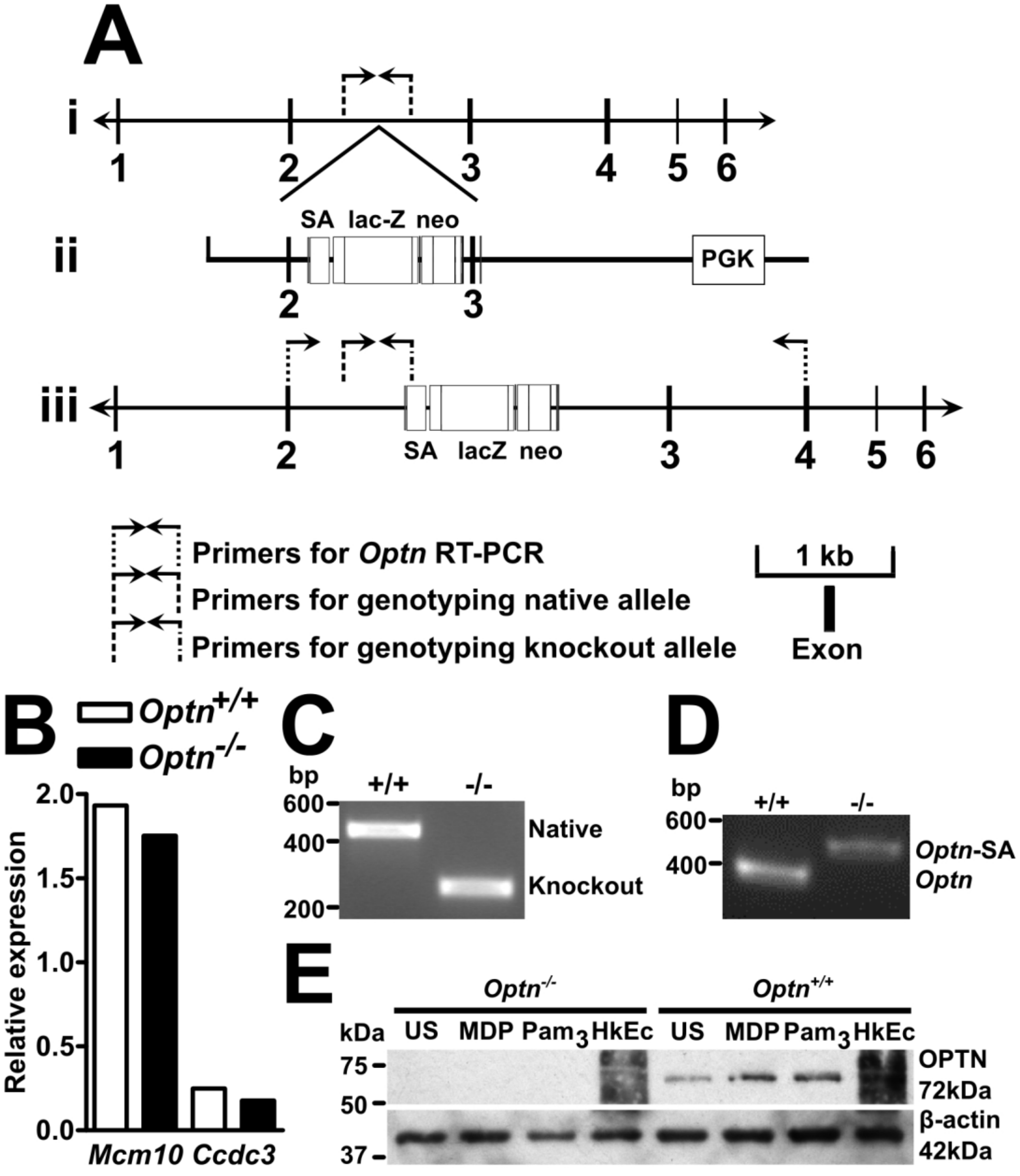

Figure S5 OPTN knockout mouse generation and verification. (a) The exon-intron layout of the murine *Optn* gene on chromosome 2 (i), structure of the L1/L2 human β-actin promoter driven neoR targeting cassette with a PGK backbone (ii) and the *Optn* locus after homologous recombination (iii). Positions of primers used for genotyping and RT-PCR are depicted, widths of exons, introns and targeting cassette are proportional to sequence length. (b) qRT-PCR of *Mcm10* and *Ccdc3* that flank *Optn* on chromosome 2 in

HkEc stimulated *Optn*$^{+/+}$ and *Optn*$^{-/-}$ BMDM were performed. (c) Genotyping of *Optn*$^{+/+}$ and *Optn*$^{-/-}$ mice was performed by PCR of gDNA isolated from ear clips amplified with *Optn* gene-specific primers, producing a 238 bp knockout amplicon and a 435 bp native amplicon. (d) RT-PCR of *Optn*$^{+/+}$ and *Optn*$^{-/-}$ BMDM total RNA produced a larger *Optn*-SA amplicon from the *Optn*$^{-/-}$ mouse containing an additional 115 bp splice acceptor (SA) sequence, which contained multiple premature termination codons on sequencing. (e) Immunoblot of thioglycollate-induced peritoneal macrophage whole cell lysates confirms the lack of OPTN protein in the *Optn*$^{-/-}$ mouse and upregulation of OPTN in macrophages from *Optn*$^{+/+}$ upon stimulation with MDP, Pam_3 and HkEc, shown with β-actin loading control. SA, Engrailed homeobox 2 splice acceptor; lacZ, β-galactosidase; neo, human β-actin promoter driven neomycin phosphotransferase; PGK, phosphoglycerate kinase 1 promoter backbone.

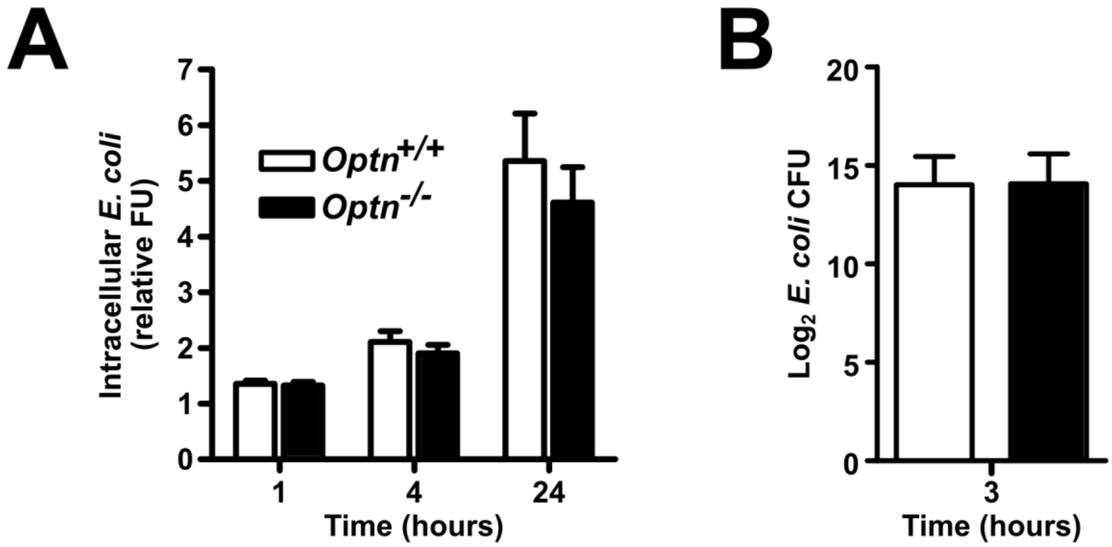

Figure S6 OPTN deficiency does not affect phagocytosis or killing of *E. coli*. (a) *Optn*$^{+/+}$ and *Optn*$^{-/-}$ BMDM were exposed to FITC-labelled HkEc then trypan blue at different time points to quantify the uptake of intracellular HkEc that is protected from quenching by extracellular trypan blue (n=5 mice/genotype over 5 experiments). (b) *Optn*$^{+/+}$ and *Optn*$^{-/-}$ BMDM were exposed to live *E. coli* for 2 hours then fresh media containing gentamicin for 1 hour to kill extracellular bacteria prior to lysing the BMDM and plating for live intracellular *E. coli* (n=3 mice/genotype over 3 experiments). Results shown are mean ± SEM (two-tailed, unpaired *t* test).

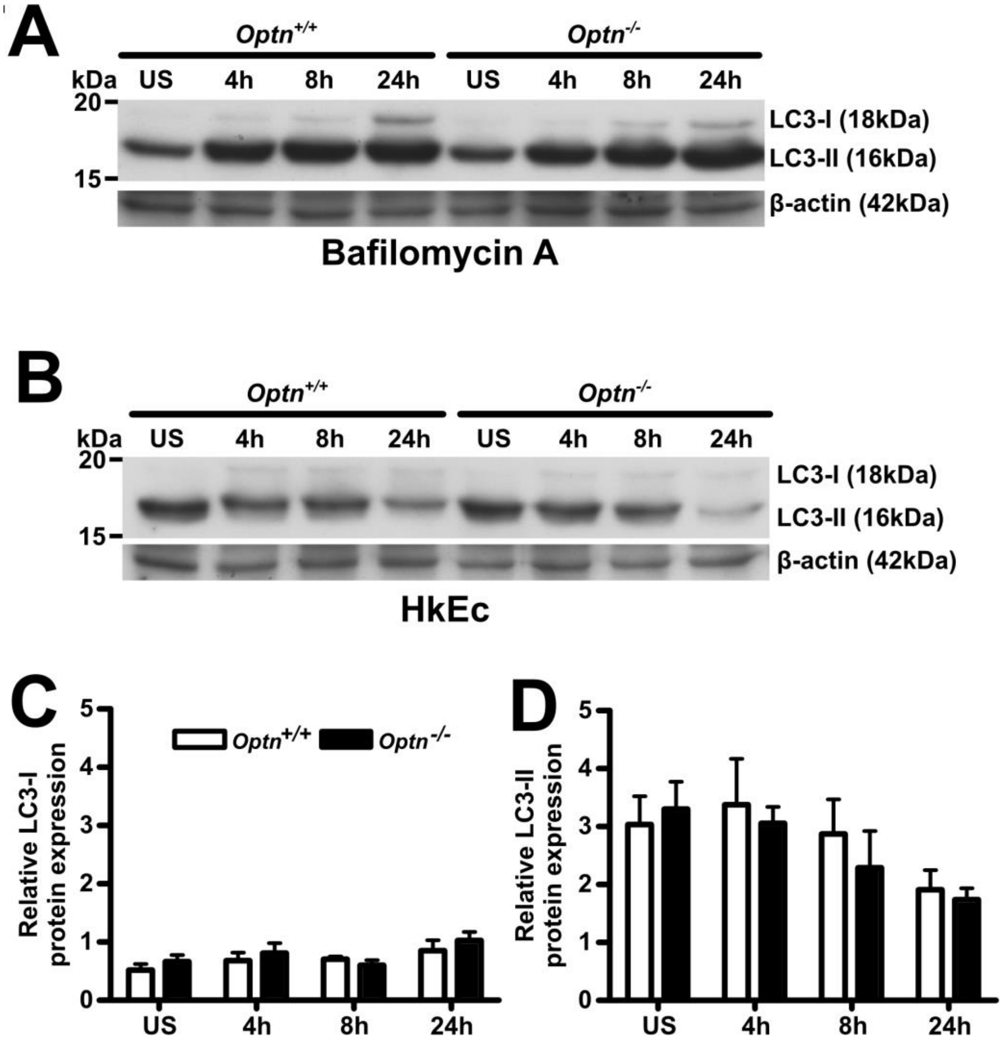

Figure S7 OPTN deficiency does not affect autophagy induction in BMDM exposed to *E. coli*. (a) *Optn*$^{+/+}$ and *Optn*$^{-/-}$ BMDM were incubated in media containing bafilomycin-A and harvested at different time points then immunoblotted for LC3B-I and LC3B-II to assess baseline autophagic flux. (b) Representative immunoblot for LC3B in *Optn*$^{+/+}$ and *Optn*$^{-/-}$ BMDM exposed to HkEc. Quantification of immunoblots for (c) LC3B-I and (d) LC3B-II in *Optn*$^{+/+}$ and *Optn*$^{-/-}$ BMDM exposed to HkEc at different time points (n=5-6 mice/genotype over 3 experiments). Results shown are mean ± SEM, all immunoblots are normalised to actin (two-tailed, unpaired *t* test).

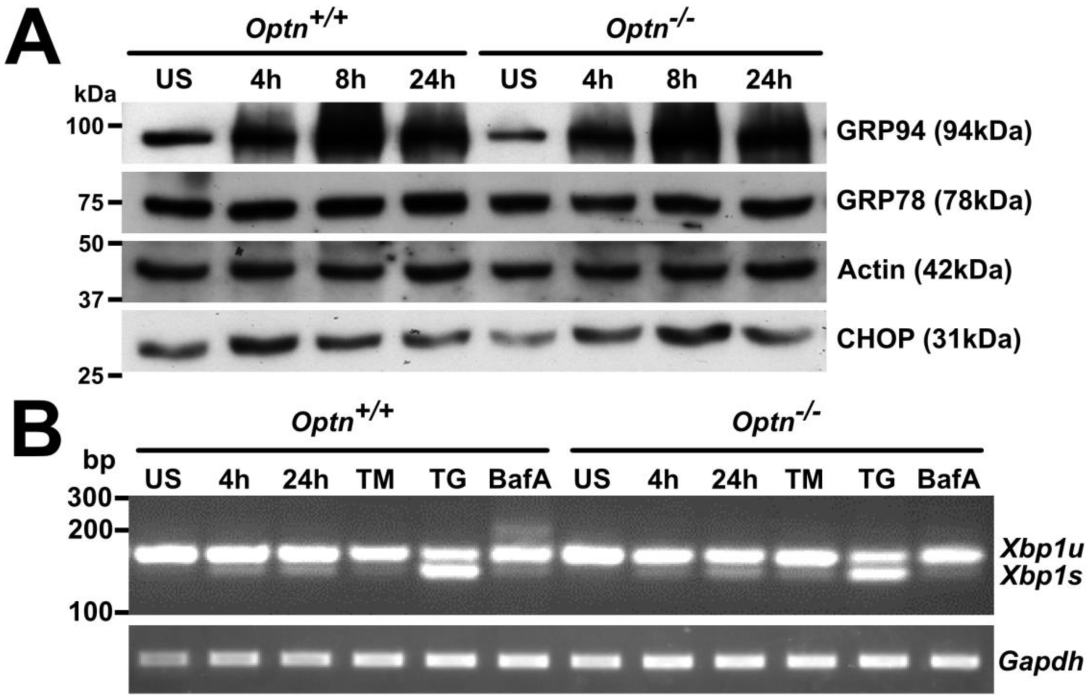

Figure S8 OPTN deficiency does not result in endoplasmic reticulum (ER) stress in BMDM exposed to *E. coli*. (a) Immunoblot for ER stress markers GRP94, GRP78/BiP and CHOP in *Optn*$^{+/+}$ and *Optn*$^{-/-}$ BMDM exposed to *E. coli* at different time points. (b) *Xbp1* splicing on PCR of *Optn*$^{+/+}$ and *Optn*$^{-/-}$ BMDM that were unstimulated (US), exposed to *E. coli* for 4 hours, 24 hours, tunicamycin, thapsigargin and bafilomycin-A for 4 hours.

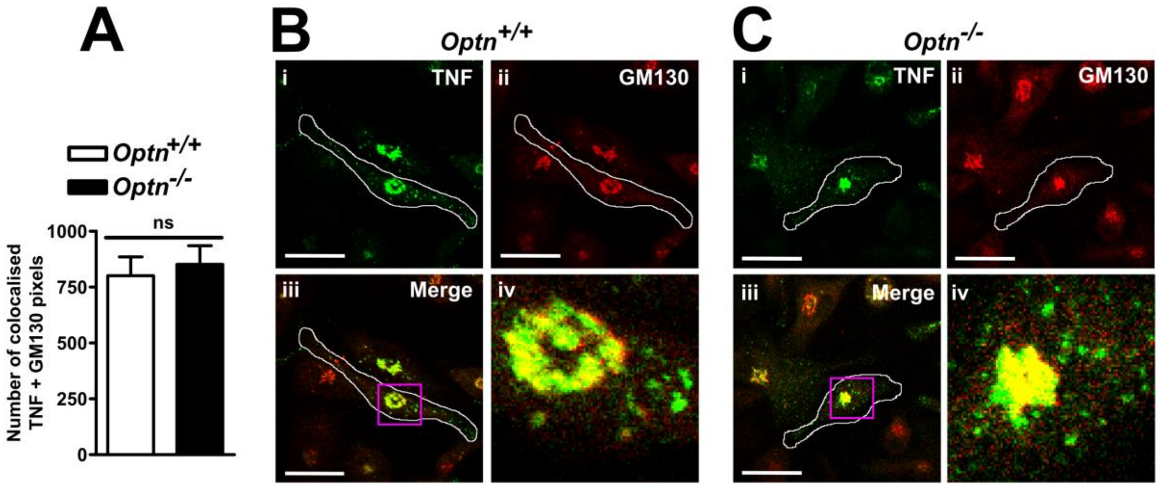

Figure S9 TNF localizes to the Golgi complex in BMDM. (a) Quantification of colocalised pixels of TNF and GM130 in *Optn*$^{+/+}$ and *Optn*$^{-/-}$ BMDM using ImageJ. Confocal microscopy images of BMDM (white outline) from (b) *Optn*$^{+/+}$ and (c) *Optn*$^{-/-}$ mice stimulated with HkEc for 4 hours and stained for (i) TNF and (ii) GM130. Merged images (iii and iv) reveals colocalisation of GM130 with TNF in the Golgi complex (image i, ii, iii at 63×, scale bar 20 µm; n= 35 cells/genotype). Results shown are mean ± SEM (ns, non-significant; two-tailed, unpaired *t* test).

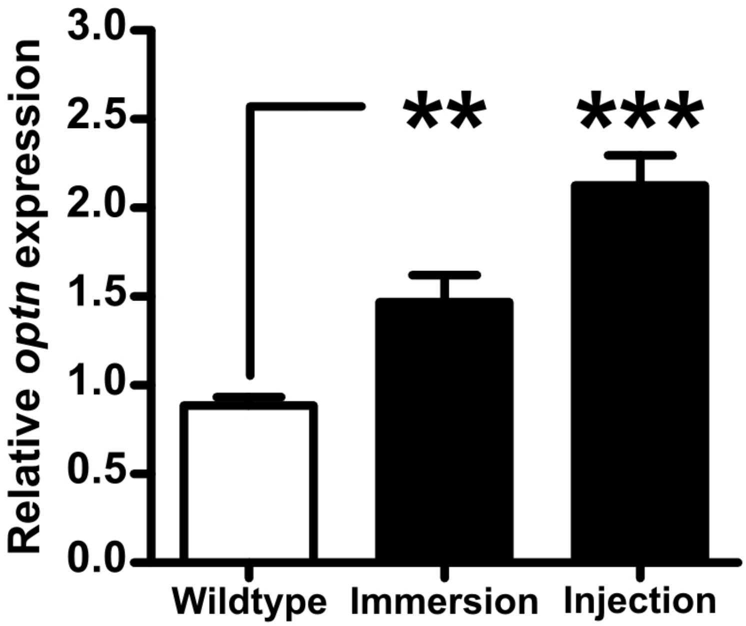

Figure S10 Zebrafish *optn* expression was elevated after infection with *Salmonella*. *Optn* was quantified using qRT-PCR in unstimulated wildtypes and after (bath) immersion and injection infection of *Salmonella* at 24 hours (n=15-32 zebrafish/group over 2-5 experiments). Results shown are mean ± SEM (**p<0.01 and ***p<0.001; one-way ANOVA and Bonferroni's test for multiple comparisons).

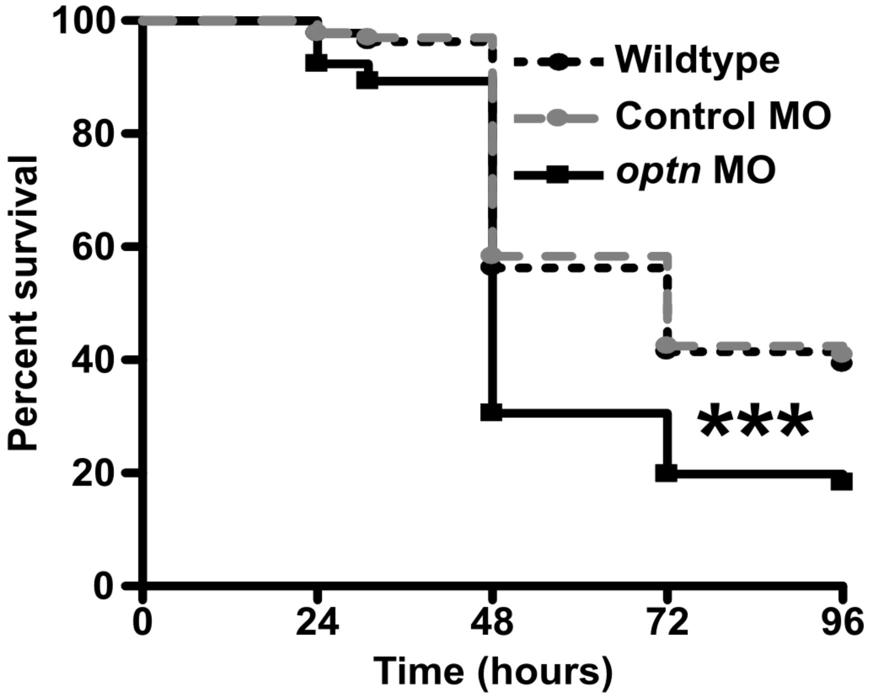

Figure S11 O*ptn* is protective against *Salmonella* infection in zebrafish. *Optn* and control morpholino injected zebrafish embryos at the one to four-cell stage were injected with *Salmonella* at 2 dpf and followed for 96 hours (n=104–119 zebrafish/group over 2 experiments). Results shown are mean ± SEM ***p<0.001; logrank test).

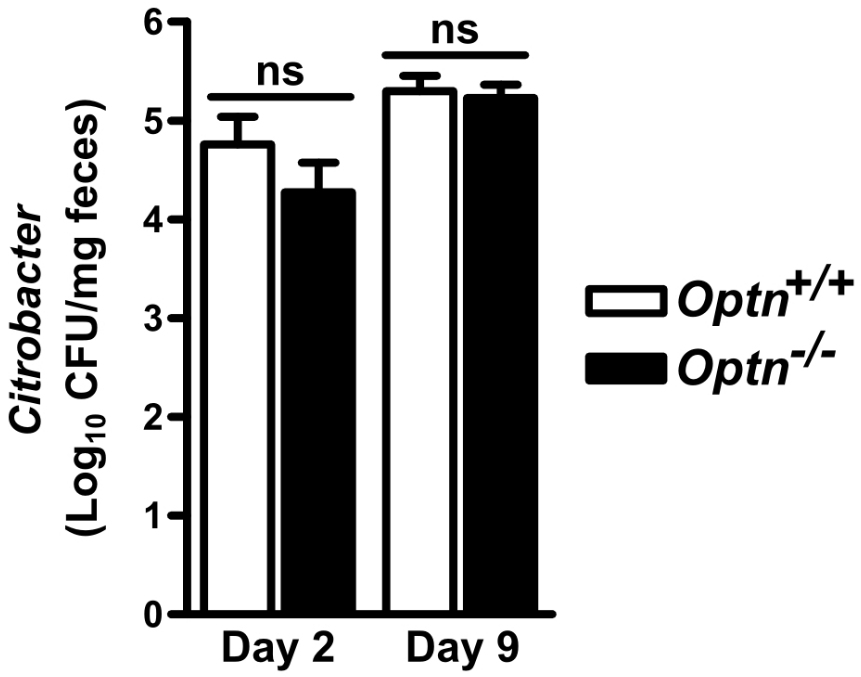

Figure S12 Faecal *Citrobacter* levels in *Optn*$^{+/+}$ and *Optn*$^{-/-}$ mice is no different. Faecal culture for *Citrobacter* at day 2 and day 9 of inoculation corrected to faecal weight in *Optn*$^{+/+}$ and *Optn*$^{-/-}$ mice. Results shown are mean ± SEM (ns, non-significant; two-tailed, unpaired *t* test).

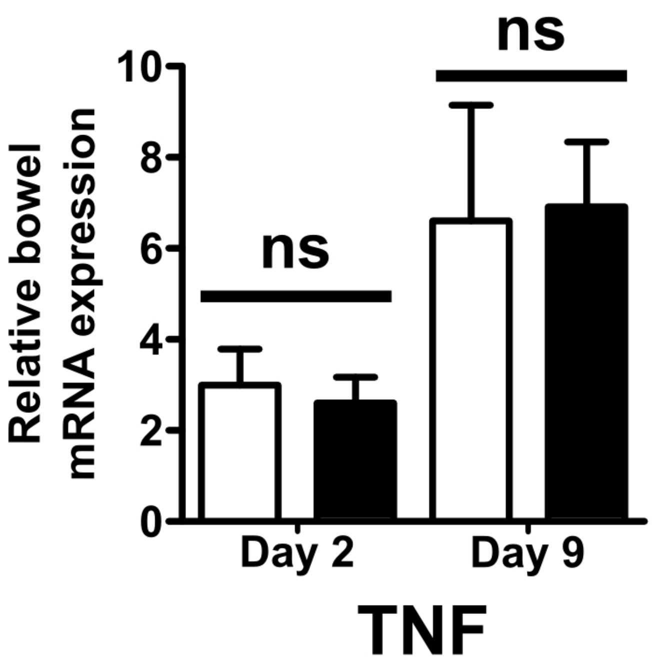

Figure S13 *Tnf* expression is the same in *Optn*$^{+/+}$ and *Optn*$^{-/-}$ large bowel in *Citrobacter* colitis. qRT-PCR of large bowel *Tnf* expression at day 2 and day 9 after *Citrobacter* inoculation. Results shown are mean ± SEM (ns, non-significant; two-tailed, unpaired *t* test).

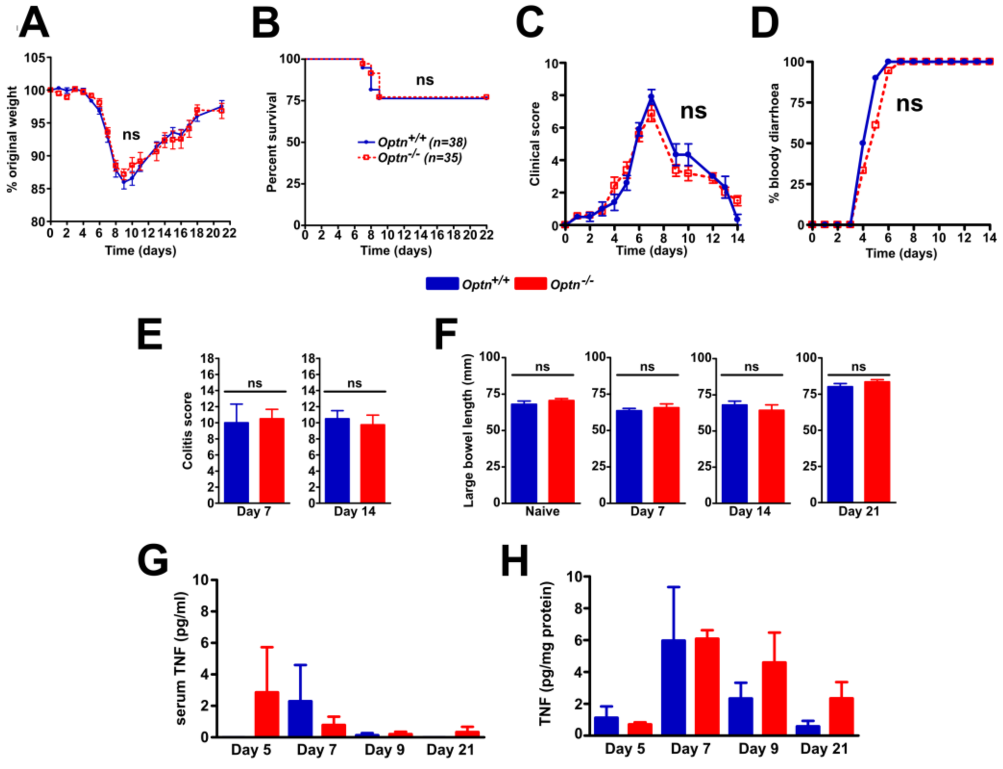

Figure S14 OPTN deficient mice do not exhibit a more severe dextran sodium sulphate (DSS) phenotype. *Optn*$^{+/+}$ and *Optn*$^{-/-}$ mice were fed 2% DSS in their drinking water and were monitored daily for (a) weight loss (n=35-38 mice/genotype), (b) mortality (n=35-38 mice/genotype), (c) clinical scores (n=10-20 mice/genotype) or (d) haemorrhagic diarrhoea (n=10-20 mice/genotype). (e) Blinded colitis scoring on H&E sections were performed during maximal insult with DSS at day 7 and recovery at day 14 (n=3-5 mice/genotype). (f) Large bowel length was measured in naïve mice, at day 7, day 14 and day 21 after commencing DSS (n=4-9 mice/genotype). (g) Serum TNF was measured at day 5, day 7, day 9 and day 21 after starting DSS (n=4 mice/genotype). (h) Bowel TNF was measured in homogenised large bowel at day 5, day 7, day 9 and day 21 and corrected to total protein extracted (n=4 mice/genotype). Results shown are mean ± SEM (ns, non-significant; two-tailed, unpaired *t* test).

SUPPLEMENTARY REFERENCES

Skarnes, W. C., Rosen, B., West, A. P., Koutsourakis, M., Bushell, W., Iyer, V., Mujica, A. O., Thomas, M., Harrow, J., Cox, T. et al. (2011). A conditional knockout resource for the genome-wide study of mouse gene function. *Nature* **474**, 337-342.

Smith, A. M., Sewell, G. W., Levine, A. P., Chew, T. S., Dunne, J., O'Shea, N. R., Smith, P. J., Harrison, P. J., Macdonald, C. M., Bloom, S. L. et al. (2015). Disruption of macrophage pro-inflammatory cytokine release in Crohn's disease is associated with reduced optineurin expression in a subset of patients. *Immunology* **144**, 45-55.
